# A comprehensive transcriptomic analysis of the bisphenol A affected kidney in mice

**DOI:** 10.3389/fmolb.2023.1260716

**Published:** 2023-11-24

**Authors:** Marta Wiszpolska, Ewa Lepiarczyk, Łukasz Paukszto, Karol Gustaw Makowczenko, Aleksandra Lipka, Mateusz Artur Maździarz, Iwona Polak, Krystyna Makowska, Sławomir Gonkowski, Paulo Correia-de-Sá, Marta Majewska

**Affiliations:** ^1^ Department of Human Physiology and Pathophysiology, School of Medicine, University of Warmia and Mazury in Olsztyn, Olsztyn, Poland; ^2^ Department of Botany and Nature Protection, Faculty of Biology and Biotechnology, University of Warmia and Mazury in Olsztyn, Olsztyn, Poland; ^3^ Department of Reproductive Immunology and Pathology, Institute of Animal Reproduction and Food Research of PAS, Olsztyn, Poland; ^4^ Institute of Oral Biology, Faculty of Dentistry, University of Oslo, Oslo, Norway; ^5^ Department of Biochemistry, Faculty of Biology and Biotechnology, University of Warmia and Mazury in Olsztyn, Olsztyn, Poland; ^6^ Department of Clinical Diagnostics, Faculty of Veterinary Medicine, University of Warmia and Mazury in Olsztyn, Olsztyn, Poland; ^7^ Department of Clinical Physiology, Faculty of Veterinary Medicine, University of Warmia and Mazury in Olsztyn, Olsztyn, Poland; ^8^ Laboratório de Farmacologia e Neurobiologia, Center for Drug Discovery and Innovative Medicines (MedInUP), Instituto de Ciências Biomédicas de Abel Salazar, Universidade do Porto, Porto, Portugal

**Keywords:** bisphenol A, kidney, oxidative stress, RNA-Seq, mitochondrial dysfunction

## Abstract

**Introduction:** Bisphenol A (BPA) is a substance belonging to the endocrine-disrupting chemicals, globally used in the production of polycarbonate plastics. It has been found that BPA enhances carcinogenesis, triggers obesity and exerts a pathogenic effect in several disorders, such as type 2 diabetes, asthma, or increased blood pressure. Recent studies have revealed, that BPA has a harmful impact on the kidneys function, therefore, the current research aimed to explore the specific molecular changes triggered in these organs after oral BPA exposure in mice.

**Materials and Methods:** The experiment was carried out on 12 (3-month-old) female mice. Six mice served as controls. The other 6 mice were treated with BPA in the drinking water at a dose of 50 mg/kg b. w. for 3 months. Then animals were euthanized, the kidneys were collected, and extracted RNA was used to perform RNA-seq.

**Results:** Applied multistep bioinformatics revealed 433 differentially expressed genes (DEGs) in the BPA-treated kidneys (232 upregulated and 201 downregulated). Additionally, 95 differentially expressed long-noncoding RNAs (DELs) were revealed in BPA samples. The Gene Ontology (GO) and Kyoto Encyclopedia of Genes and Genomes (KEGG) annotations indicated that BPA exposure resulted in profound changes in several essential processes, such as oxidative phosphorylation, mitochondrial and ribosome function, or chemical carcinogenesis.

**Conclusion:** The obtained novel results suggest that BPA has a harmful impact on the fundamental processes of the kidney and significantly impairs its function by inducing mitochondrial dysfunction leading to oxidative stress and reactive oxygen species production.

## 1 Introduction

Bisphenol A (BPA), 2,2-bis(4-hydroxyphenyl)propane, is a product of the condensation reaction of two molecules of phenol and one molecule of acetone, in the presence of hydrogen chloride (Ma et al., 2019). BPA, according to the U.S. Environmental Protection Agency, belongs to the endocrine-disrupting chemicals (EDCs), which are responsible for reproductive and developmental dysfunctions in laboratory rats (Alonso-Magdalena et al., 2016). EDCs are naturally occurring or synthetic exogenous substances capable of disturbing the homeostasis of the human endocrine system, thus affecting physiological functions such as growth, metabolism and reproduction (Alonso-Magdalena et al., 2016). Recent researches prove that EDSs cause diseases such as diabetes, cancer, fatty liver disease, neurological disorders, and dysfunctions of the reproductive system in both women and men (Sukjamnong et al., 2020).

In humans, the main exposure to BPA comes from food and water, primarily due to the direct contact with food containers and other materials used in the course of production, handling, and transportation (Moreno-Gómez-Toledano et al., 2021). Another important source of BPA exposure is air. Household items such as epoxy flooring, adhesives or electronic equipment may release BPA which accumulates in dust and is absorbed by the respiratory tract (Ma et al., 2019). BPA is also used in the chemical production of polycarbonate plastics which can be found in healthcare equipment (dental composites, contact lenses), products for children (toys, soothers, milk bottles), a thermal paper that is used in receipts and even cinema tickets (Konieczna et al., 2015). The National Health and Nutrition Examination Survey performed between 2005 and 2010 revealed that in humans the average daily intake of BPA is in the range of 0.03–0.07 μg/kg b.w./day (Lorber et al., 2015).

Due to the phenolic structure, BPA can interact with estrogen receptors and interfere with endogenous hormones by binding to transporter proteins and affecting their free and bound levels in the plasma. BPA exerts a pathogenic effect in several endocrine disorders and affects both female and male reproductive systems (Ma et al., 2019). Moreover, it has been found that contact with BPA triggers obesity, type 2 diabetes, asthma, increased blood pressure, and developmental diseases such as reduced birth weight or shortened anogenital distance. It has been proven that BPA can modify the epigenome, and thus influences disease susceptibility (Alonso-Magdalena et al., 2016). Furthermore, BPA exposure enhances carcinogenesis and inflammatory or immune responses (Brandt et al., 2014).

BPA metabolism takes place mainly in the liver, where it is broken down by enzymes such as UDP-glucuronosyltransferase 2B15 (UGT2B15) and estrogen sulphotransferase. Therefore in our previous study, we have investigated changes in the liver transcriptomics of mice exposed to BPA in drinking water. Data from this study suggested that BPA has a significant impact on gene expression in this organ, and may alter pathways linked to the pathogenesis of severe metabolic liver disorders and malignant tumors, in particular hepatocellular carcinoma (Wiszpolska et al., 2023). About 1% of BPA does not break down and accumulates in tissues. However, most conjugates generated by BPA with glucuronides and sulfates are excreted into urine or faeces after a few days (Michałowicz, 2014). Recent studies have revealed, that BPA has a detrimental effect on kidneys and contributes to the progression of kidney damage by enhancing oxidative stress, inducing an inflammatory response, blocking autophagic flow, and exacerbating tubular damage, thus leading to excessive collagen accumulation and renal fibrosis (Priego et al., 2021).

Taking into account the existing knowledge, the present study was designed to provide detailed information on the influence of oral exposure to BPA on the transcriptomic profile of kidneys in mice. To achieve this goal a targeted bioinformatic analysis of the control and BPA-treated kidney samples was performed to explore the specific molecular pathways that underlie the BPA mechanism of action. The discovery of specific dysregulated genes may shed new light on the full impact of this chemical on the affected organs.

## 2 Materials and methods

### 2.1 Laboratory animals

The experiment was carried out on twelve, 3-month-old female mice (*Mus musculus*, C57BL6/J/CMDB strain) with an average body weight of 30 g. The same animals were used in our previous study to investigate changes in the liver transcriptomics of mice exposed to BPA in drinking water (Wiszpolska et al., 2023). Mice were kept in the animal house (at the Faculty of Veterinary Medicine, University of Warmia and Mazury in Olsztyn, Poland) under constant temperature 22°C ± 20°C, humidity 55% ± 10% and 12:12 h light-dark cycle. The animals had free access to food and water. The study was conducted according to the guidelines of the Local Ethics Committee for Animal Experimentation in Olsztyn, Poland (affiliated with the National Ethics Committee for Animal Experimentation, Polish Ministry of Science and Higher Education; decision No. 46/2019). The animals were randomly divided into two groups. Six mice served as controls (CTR) and were not subjected to any experimental procedures. The other 6 mice served as an experimental group and were treated with BPA in the drinking water at a dose of 50 mg/kg b.w. for 3 months–which is considered a Lowest Observed Adverse Effect Level (LOAEL) for this species (Choi et al., 2010). Both groups of mice were given the same food, they were also weighed weekly, and the BPA dose was gradually increased on this basis. After 3 months, the animals were sacrificed by decapitation (Makowska et al., 2022). Immediately after death, the kidneys were removed under sterile conditions, then placed in liquid nitrogen and stored at −80°C until further analysis.

### 2.2 RNA extraction, library construction and high-throughput transcriptome sequencing

The total RNA of both groups was isolated from kidneys using the mirVanaTM miRNA Isolation Kit with phenol according to the manual (Thermo Fisher Scientific, United States). A 2100 Bioanalyzer (Agilent Technologies, United States) with a 6000 Nano LabChip Kit was used to measure the quantity and quality of total RNA isolates. Only the samples with the highest RIN values (greater than or equal to 7.5) and concentrations were selected for RNA-Seq library construction. The sequencing procedure was held by an outsourcing company (Macrogen, South Korea) exploiting Illumina NovaSeq 6000 System (Illumina, San Diego, CA, United States). Briefly, 1 µg of total RNA for each sample was selected for library construction by the Illumina TruSeq mRNA LT Sample Prep Kit (Illumina, Inc., San Diego, CA, United States). The first step involved the purification of mRNA molecules using poly-T-attached magnetic beads. Next, the mRNA was cut into small fragments with divalent cations. The cleaved RNA pieces were amplified into the first-strand cDNA using SuperScript II reverse transcriptase (Invitrogen, Waltham, MA, United States) and random primers. In the upstream step, second-strand cDNA synthesis using DNA Polymerase I and RNase H was performed. The purified products of PCR reactions were enriched and the final cDNAs libraries were constructed. The RNA-seq libraries were quantified using qPCR according to the qPCR Quantification Protocol Guide (KAPA Library Quantification kits for Illumina Sequencing platforms) and qualified using the TapeStation D1000 ScreenTape (Agilent Technologies, Waldbronn, Germany). Indexed libraries were then sequenced using the NovaSeq6000 platform (Illumina, San Diego, CA, United States).

### 2.3 In silico profiling of kidney transcriptome affected by BPA

#### 2.3.1 Raw reads processing and mapping to a reference genome

The raw high-throughput sequencing dataset retrieved from NovaSeq 6000 was evaluated according to the quality control standards using FastQC software version 0.11.7 (Andrews, 2010). The paired-end reads (2 × 150 bp, type stranded) were trimmed after the Illumina adaptors identification within the sequences and also low-quality reads (PHRED cut-off score <20) were excluded from downstream analysis using Trimmomatic software v. 0.38 (Bolger et al., 2014). The 120 bp trimmed reads were mapped to the mouse reference genome according to ENSEMBL annotation (Mus_musculus.GRCm39), using STAR software v. 2.7.10a (Dobin et al., 2013). Sequences aligned multiple times were not considered for subsequent analysis. The StringTie v. 2.2.1 pipeline was incorporated to re-evaluate the ENSEMBL annotations to obtain novel annotations of intergenic-expressed regions (Pertea et al., 2015). The integrity of the RNA-seq libraries was validated and clustered with the ggplot2 Bioconductor library v. 3.3.5 of R software v. 4.1.3 (R Core Team; https://cran.r-project.org/) (Ramos et al., 2017). Whole transcriptome high-throughput sequencing (RNA-seq) of BPA libraries was applied to identify expression profiles of differentially expressed genes (DEGs), differentially expressed long non-coding RNA (DELs) and differential alternative splicing events (DASs).

#### 2.3.2 Detection of differentially expressed genes and long non-coding RNAs and interaction analyses

The differentially expressed analyses were performed for the protein-coding transcripts. The expressed transcripts were grouped according to the genomic localization and tagged as transcriptionally active regions (TARs). The differentially expressed analysis was performed by the DESeq2 tool v. 1.36.0 (Love et al., 2014), with a negative binomial generalized linear model implemented. Only TARs whose expression modification patterns reached the presumed binary logarithm of fold change (log2FC) cutoff level (absolute log2FC > 1) and significance threshold (adjusted *p*-value <0.05) were included in further analyses. TARs located on the reference genome within the range of protein-coding genes were classified as DEGs, while those occurring in regions of long non-coding RNAs were assigned to DELs. Relationships between DEGs and DELs were estimated by co-expression analysis. DELs−DEGs pairs located on different chromosomes, but showing similarity of transcriptional profiles, were characterized as *trans*-interactions based on Pearson’s correlation coefficient (absolute r value >0.9 and *p*-value <0.05). For *cis* interactions between DEGs−DELs the FEELnc software was used (Wucher et al., 2017).

#### 2.3.3 Differential alternative splicing events analysis

Alternative splicing events were predicted using the rMATS tool v. 4.1.0 (Shen et al., 2014) based on StringTie’s output files. DASs between BPA and CTR murine groups were statistically tested and the inclusion level difference for all splicing events was estimated. Detected DASs were considered statistically significant with a false discovery rate (FDR) < 0.001, and the absolute value of the percentage of splicing inclusions difference (ΔPSI) > 0.1. All discovered DASs were classified into five categories by rMATS: alternative 5′ splice site (A5SS), alternative 3′ splice site (A3SS), mutually exclusive exons (MXE), retained intron (RI) and skipping exon (SE). DASs involved in relevant physiological processes identified during functional analyses were visualized using the rmats2sashimiplot Python tool v. 2.0.4 and the Circos software v. 0.69–9 (Krzywinski et al., 2009).

#### 2.3.4 Functional annotations of DEGs, DELs and DASs

Obtained DEGs, DELs and DASs were scanned using g:Profiler software (Reimand et al., 2016) against Gene Ontology Consortium (GO) (Ashburner et al., 2000; Carbon et al., 2017) and Kyoto Encyclopedia of Genes and Genomes (KEGG) (Kanehisa et al., 2017) annotations. The essential genes were annotated to ontological terms within three aspects, such as biological processes (BP), cellular components (CC), molecular functions (MF), and also assigned to signaling and metabolic KEGG pathways. The enrichment analysis (FDR <0.05) was applied to uncover ontology and pathway annotations regulated by DEGs, DELs and DASs. To obtain the kidney gene signatures, the DEGs were also scanned according to the Human Phenotype Ontology (HP) database following the terms representing individual phenotypic anomalies (https://hpo.jax.org/app/; accessed on 20 December 2021). To visualize the contribution of the identified DEGs, DELs and DASs to kidney function, those events were highlighted in the KEGG pathways using the Pathview v. 1.30.1 R library (Luo and Brouwer, 2013).

### 2.4 Real-time PCR

The mRNA content of selected genes was determined by Real-time PCR. Primers for the selected genes were designed using Primer3Plus software (Untergasser et al., 2012) (ELIXIR, Hinxton, Cambridgeshire, United Kingdom) based on the sequences listed in Supplementary Table S1. The cDNA was obtained using the Applied Biosystems™ High-Capacity cDNA Reverse Transcription Kit (Thermo Fisher Scientific, Vilnius, Lithuania) according to the manufacturer’s protocol. Real-time PCR was performed using the Applied Biosystems™ PowerUp™ SYBR™ Green Master Mix (Thermo Fisher Scientific, Vilnius, Lithuania) according to the manufacturer’s protocol on the QuantStudio™ 3 Real-Time PCR System (Applied Biosystems™, Thermo Fisher Scientific Inc., Waltham, MA, United States). Briefly, each reaction contained 5 μL of master mix (2X), forward and reverse primers at 1,000 nM each, 10 ng of cDNA, and an appropriate volume of nuclease-free water to a final volume of 10 μL. Reactions were performed in four technical replicates for each biological sample. Expression of each gene was calculated using the comparative Pfaffl method (Pfaffl, 2001), in which expression of the gene of interest in treated samples is represented as a fold change compared with control samples and normalized to endogenous reference genes (*UBC*, GenBank NM_019639.4 and *ACTB* GenBank NM_007393.3) (relative quantification RQ = 1). Results were expressed as means of biological replicates ± standard deviations. Statistical analysis was performed using Student’s t-test (two-sided test) in Prism 8 software (GraphPad Software Inc., San Diego, CA, United States). *p* values ⩽ 0.05 were considered statistically significant when ⩽0.0332 (*), ⩽0.0021 (**), ⩽0.0002 (***), and ⩽0.0001 (****).

## 3 Results

### 3.1 Kidney transcriptomic statistics and the abundance of expression profiles

After sequencing, 753,323,838 raw reads were obtained, globally (Supplementary Table S2). The filtration procedure removed 114,924,754 reads with a low-quality score and the trimming procedure clipped out Illumina adaptor sequences. The surviving 638,399,084 paired-end reads were mapped to the *Mus musculus* reference genome. The results of the mapping process were applied to the identification of the DEGs and DAS events. Uniquely mapped reads contained an average of 82.89% out of all processed sequences. According to the gene structure, 58.88% of paired-end reads’ nucleotides were aligned to the coding sequence (CDS), 37.88% to the untranslated regions (UTR), 2.98% to the intronic sequences, and 2.26% to the intergenic localizations. The transcriptomic data from this study have been submitted to the European Nucleotide Archive under accession No. PRJEB59035.

### 3.2 Transcriptomic differences in the kidney under BPA influence

Screening RNA-seq data for differential gene expression analysis revealed that the kidney transcriptome affected by BPA was associated with 16,551 differentially expressed TARs. Among them, 433 TARs were classified as DEGs, which encoded protein sequences (Supplementary Table S3). Under the BPA influence, 232 DEGs were upregulated and 201 downregulated. Estimated log2FC values of DEGs ranged from 7.63 (*SULT2A2*) to −10.55 (*TMEM26*). The deep transcriptome analysis revealed 95 DELs under BPA influence, within which 43 were upregulated and 52 downregulated. Computed DELs’ log2FC values ranged from 3.34 (*D630033O11Rik*) to −8.75 (*Gm27028*). According to the ENSEMBL, the most frequent biotypes of identified long-noncoding RNAs were: antisense RNA (28 DELs) and lincRNA (17 DELs).

**FIGURE 1:**
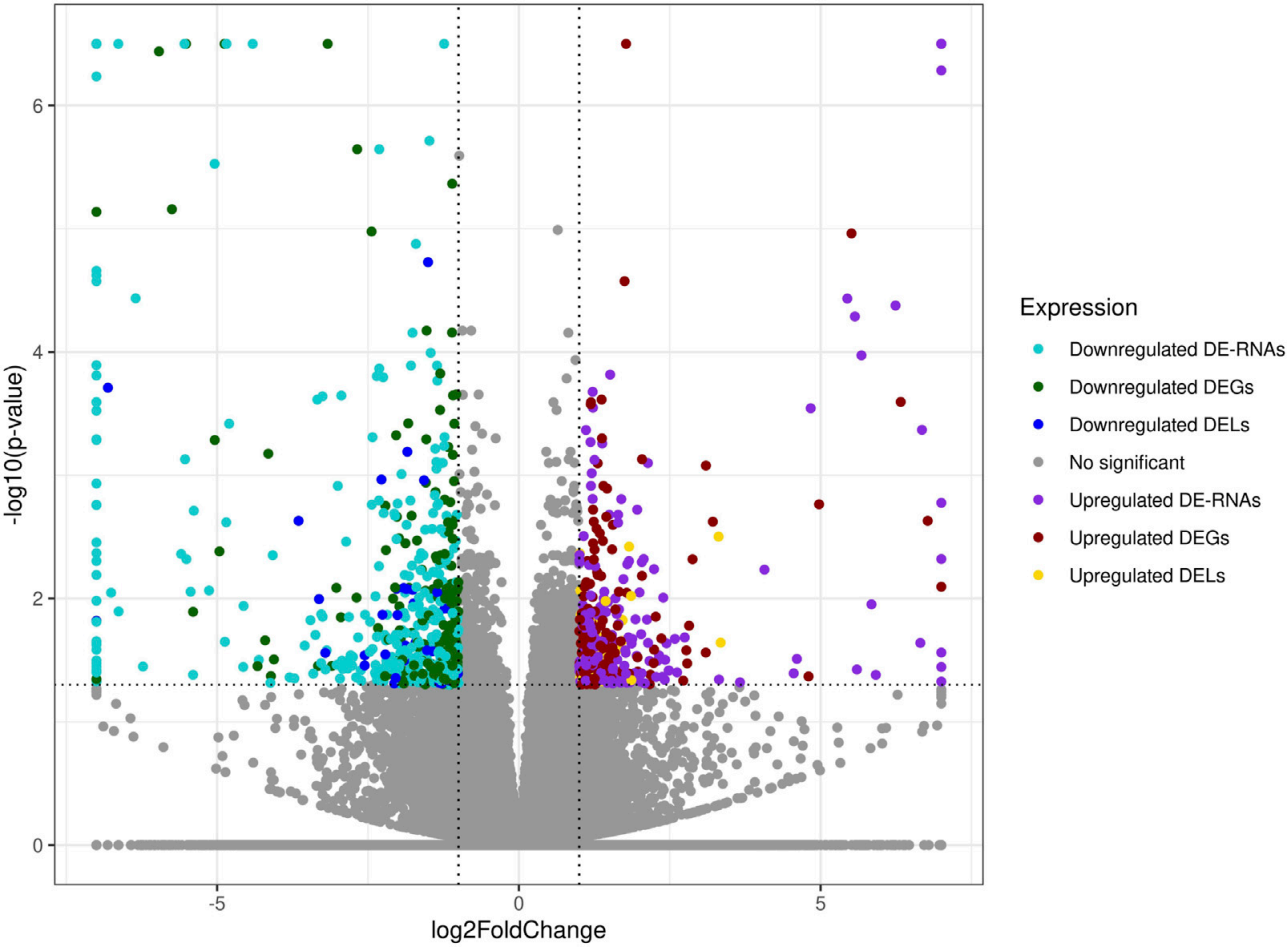
Expression profiles overview of kidney differentially expressed transcriptionally active regions (TARs) under BPA influence. Volcano plot with binary logarithmic values of fold change (log2FC; X-axis) plotted against negative logarithmic adjusted *p*-values (-log10 (*p*-value); Y-axis). The dotted horizontal line indicates a negative logarithmic adjusted *p*-value cut-off (0.05).

**FIGURE 2:**
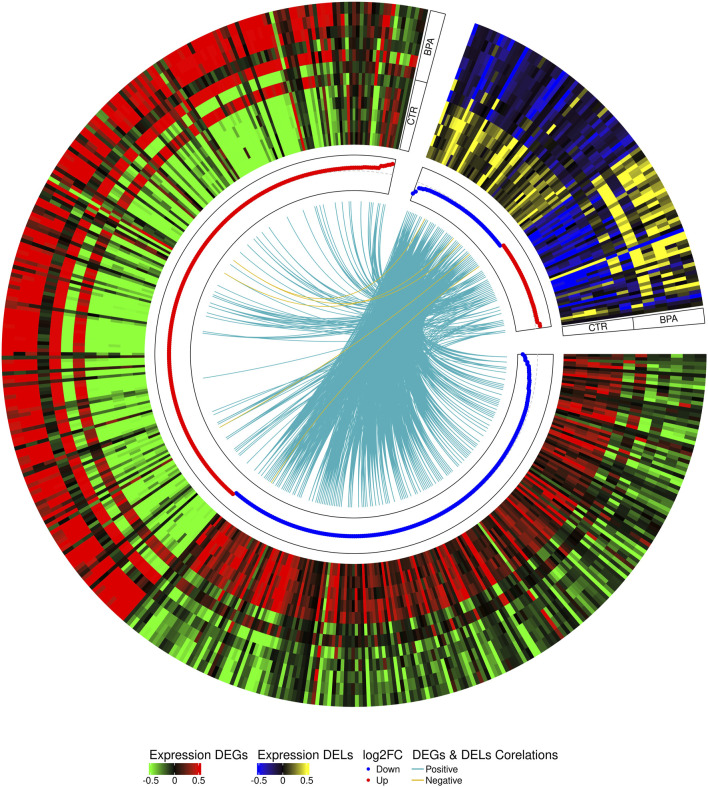
Circular heatmap visualization of differentially expressed genes (DEGs) and long non-coding RNAs (DELs) in BPA-affected and CTR-control libraries. The 12 upper tracks visualize the normalized (Z-score) expressions for DEGs in each biological replicate. The large part of the circle (green-red) depicts DEGs and the smaller part (blue-yellow) describes DELs. The inner track shows the correlation links between the co-expressed DEGs and DELs, whereas blue links depict positive (>0.9) and yellow negative (<-0.9) Pearson’s correlation.

The co-expression analysis revealed 1249 DEGs−DELs *trans*-interactions. Identified events showed the mediation of 74 DELs in the regulation of 176 DEGs (assuming a strong Pearson’s correlation; Supplementary Table S4). The majority of DEGs−DELs *trans*-actions (1244) were positively correlated, although only 5 showed a negative correlation of expression (involving 4 DEGs: *CALHM6*, *S100A4*, *TNNT1* and *ZC3HAV1L*). The expression profiles of all DEGs and DELs were presented in a volcano plot (Figure 1) and a heatmap supplemented with *trans*-interactions information (Figure 2). Moreover, 80 correlation pairs (DEGs−DELs) located in the same chromosomes have been identified and none of the DELs were localized in the vicinity (10,000 bp) of DEGs.

### 3.3 Transcriptomic alternative splicing signatures of BPA-treated kidney

**FIGURE 3:**
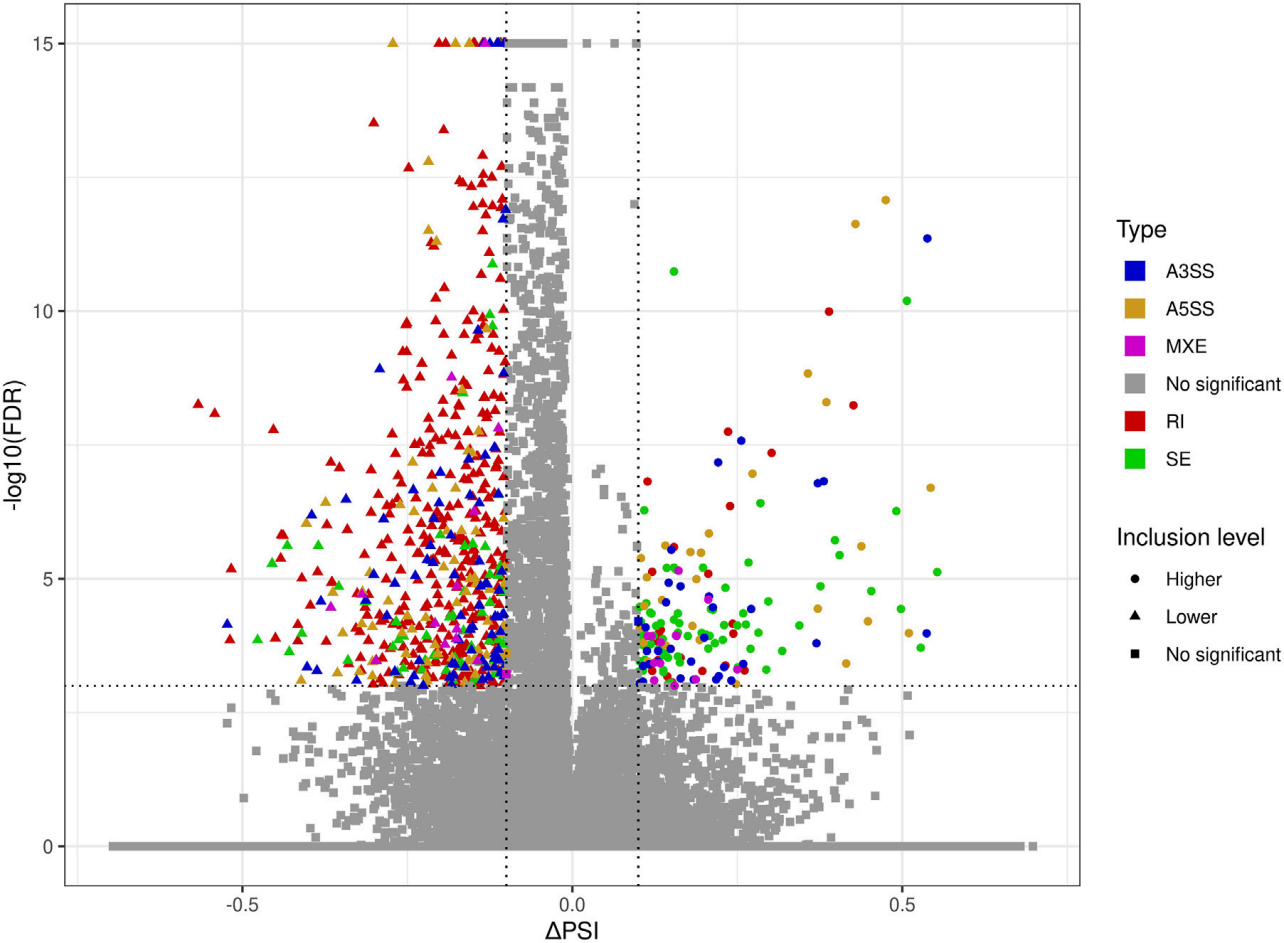
Volcano plot showing the percentage of splicing inclusions difference (ΔPSI) against the statistical significance (-log10FDR) of DASs identified within genes of murine BPA-affected kidneys vs. control samples. The dashed lines indicate the applied cut-off thresholds, described in the text.

**FIGURE 4:**
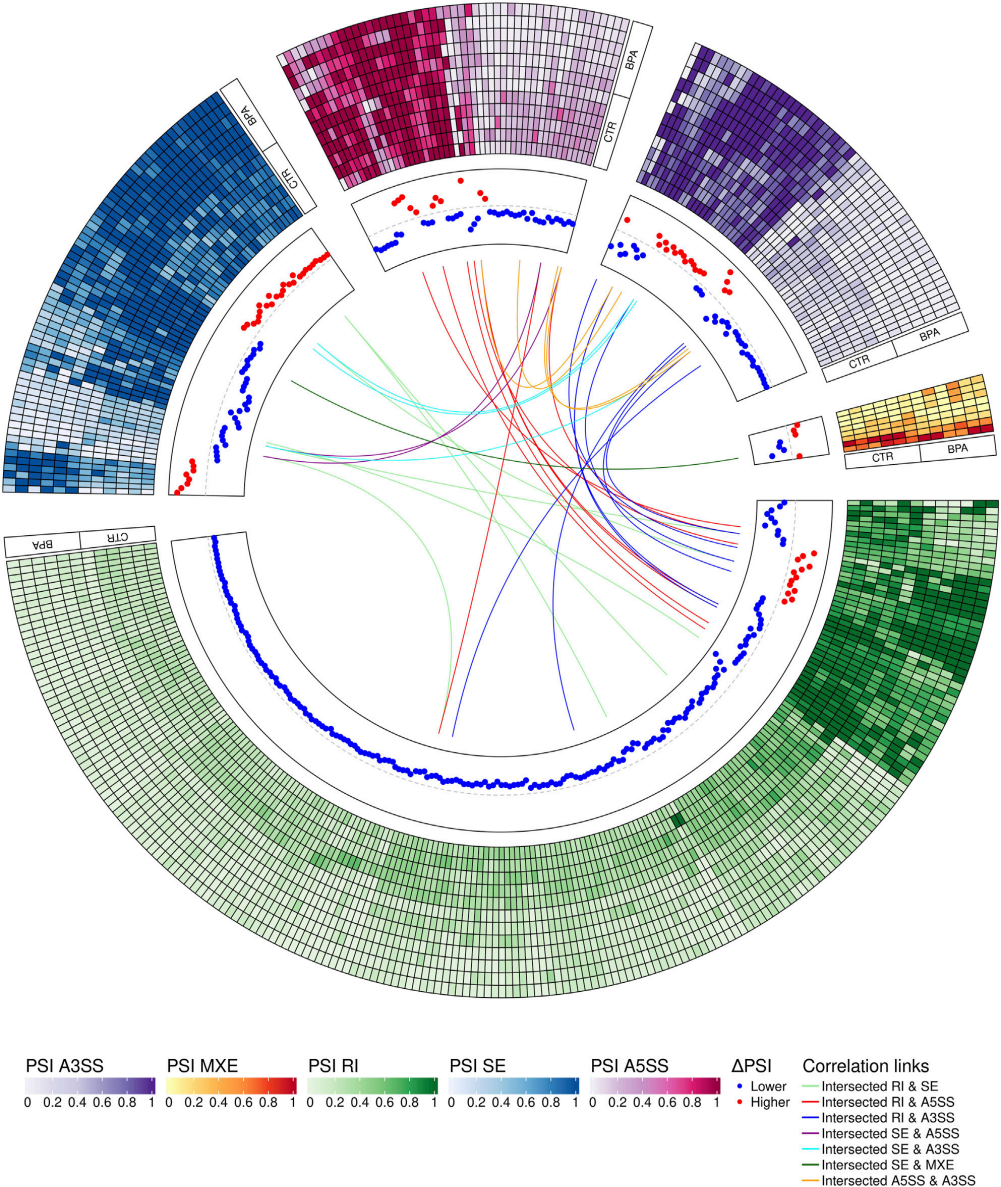
Circular visualization of differentially alternative splicing events (DASs) occurring after BPA treatment. The five-scale color heatmaps (outer track) represent percentage inclusion values (PSI) in experimental (BPA) and control (CTR) samples. The middle track shows dPSI values (red–higher inclusion level in BPA, blue–higher inclusion level in CTR). Color links join common genes with more than one DAS classified in different types of alternative splicing events.

**FIGURE 5:**
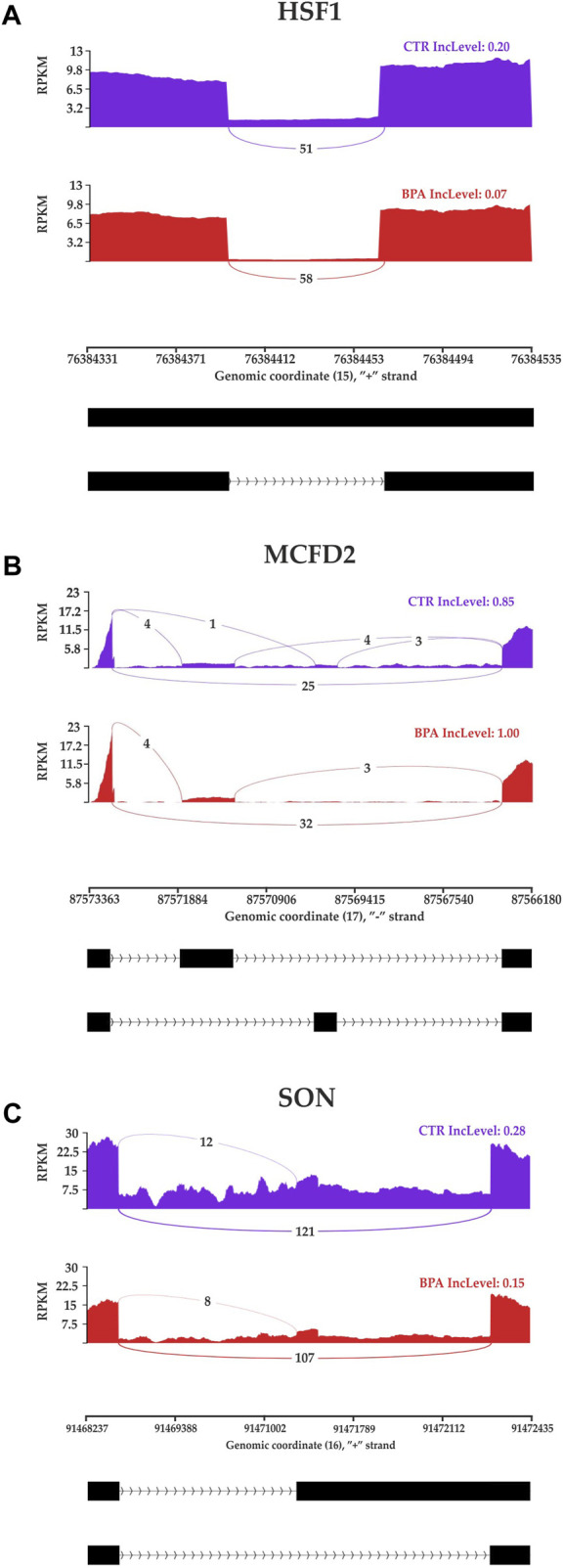
Sashimi plot visualizing the detected coverage of RNA-Seq reads on the reference genome and the average values of reads combining distant genome fragments (black blocks underneath the graphs) in CTR and BPA groups. Displayed fragments of **(A)** *HSF1*, **(B)** *MCFD2* and **(C)** *SON* genes were classified as statistically significant differentiated alternative splicing events.

The applied procedure, incorporating rMATS software, allowed the detection of 65,673 alternative splicing events, including 383 DASs resulting from the comparison of BPA vs. CTR samples (Figure 3). Among all detected DASs, 50 were classified as A5SS, 53 as A3SS, 8 as MXE, 207 as RI, and 65 as SE (Figure 4). Calculated ΔPSI values ranged from 0.54 (A3SS within *EBF1* gene) to −0.57 (RI within *Gm41792* lncRNA). All disclosed DASs were localized within 289 protein-coding genes and 12 lncRNA-coding regions. Alternative splicing events were discovered in the 12 DEGs: *ANKRD24* (SE), *CLASRP* (RI), *FUBP1* (RI), *GUK1* (RI), *HDAC7* (RI), *IL15RA* (A5SS and A3SS), *RPL13A* (RI), *TET2* (A3SS and RI), *ZKSCAN3* (RI); and 3 DELs: *Gm15860* (SE), *C030005K06Rik* (A3SS), *5031425E22Rik* (A3SS). Selected events of alternative splicing occurring within *HSF1* (RI), *MCFD2* (SE) and *SON* (A3SS) genes are visualized in Figure 5, while all identified cases are summarized in Supplementary Table S5.

### 3.4 Gene ontology networks and pathway signaling analysis of DEGs, DELs and DASs

**FIGURE 6:**
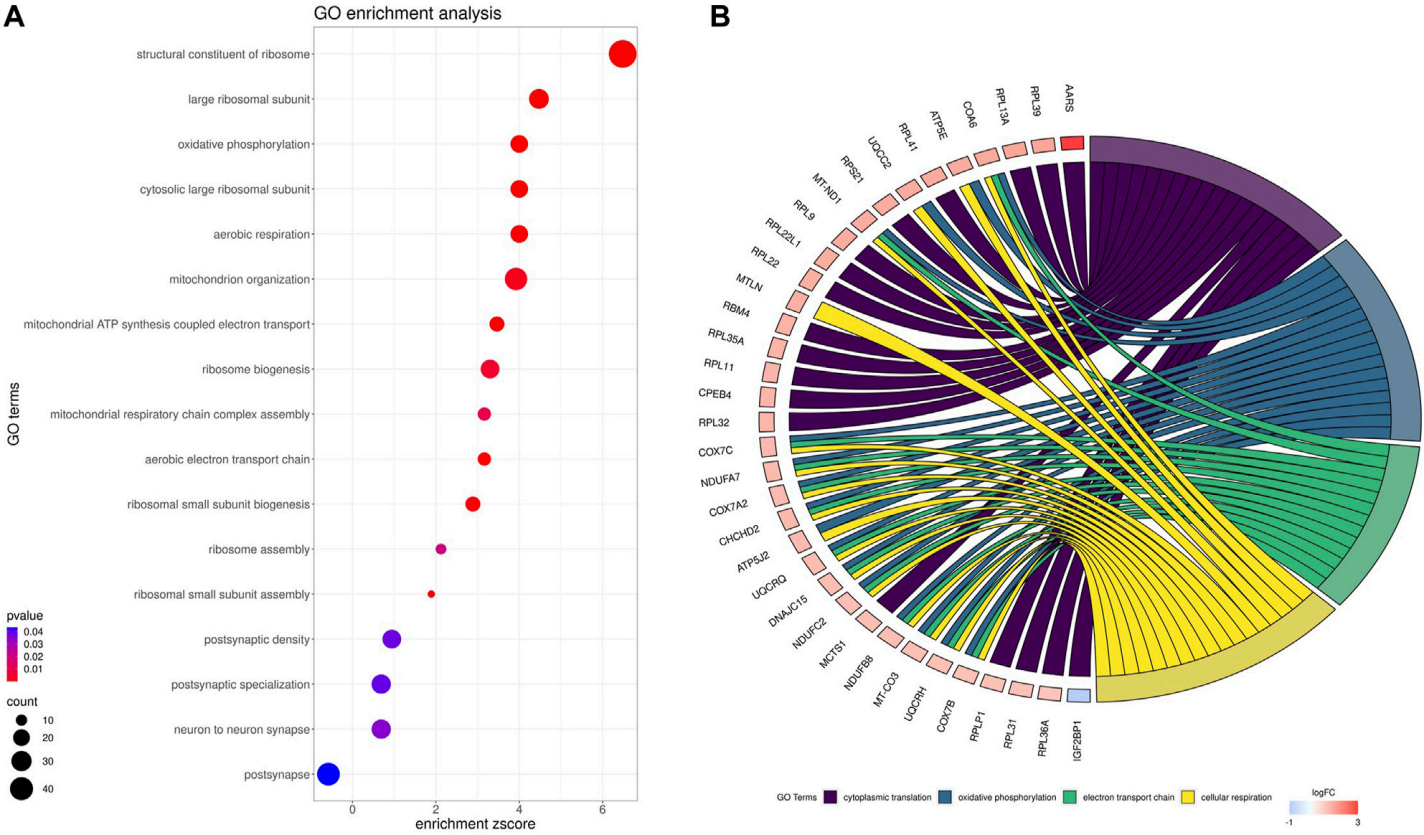
Gene Ontology (GO) enrichment dot-plot **(A)** of abundances (size of dots) and significance (color of dots) of the top GO terms. **(B)** Circos-plot relationship of differentially expressed genes (DEGs) engaged in kidney function under BPA influence significantly associated with four selected GO enriched terms. Gene symbols with logarithmic values (blue-red scale) of fold change (log2FC) are located on the left side of the circos. Color links merge genes with the GO terms (cytoplasmic translation, oxidative phosphorylation, electron transport chain and cellular respiration) on the right side.

Gene ontology (GO) enrichment analysis reflected the functional annotations of the identified TARs engaged in kidney activity under BPA impact. The 382 unique DEGs were assigned to functional GO annotations grouped into 36 BP, 5 MF and 49 CC categories (Supplementary Table S6). The GO gene annotation, enriched in BP ontological processes in BPA-affected kidneys, revealed DEGs within terms such as “Oxidative phosphorylation”, “Mitochondrial ATP synthesis coupled electron transport”, “Aerobic respiration”, “Aerobic electron transport chain”, “Mitochondrial respiratory chain complex assembly”, “Mitochondrion organization”, “Ribosomal Small Subunit Biogenesis”, “Ribosomal Small Subunit Assembly”, “Ribosome Assembly” or “Ribosome Biogenesis”. In the MF category, the DEGs were engaged in the processes involved for example in the “Structural Constituent of Ribosome”, while the CC category grouping revealed that BPA-modulated mRNAs were involved mainly in ribosome organization and function (for instance “Cytosolic large ribosomal subunit” or “Large Ribosomal Subunit”) as well as “Oxidative phosphorylation” (i.a. “Oxidoreductase complex”, “Mitochondrial respiratory chain complex I″, “NADH dehydrogenase complex”). The comprehensive GO enrichment classification was summarised in Figures 6A, B.

In the signaling pathway analysis, 11 KEGG pathways were identified for the kidney’s mRNAs modulated by BPA (Supplementary Table S6). Among the signalling and metabolic pathways, 17 DEGs enriched “Oxidative phosphorylation” (KEGG: 00190; Figure 7), 17 DEGs were found in the “Chemical carcinogenesis - reactive oxygen species” pathway (KEGG: 005208; Supplementary Figure S1), 16 DEGs were revealed in the “Parkinson disease” KEGG: 05012, Supplementary Figure S2), and 19 in the “Alzheimer disease” (KEGG: 05010; Supplementary Figure S3). The “Ribosome” (KEGG: 03010; Supplementary Figure S4) pathway mapped 33 DEGs, linked mostly to ribosomal protein (RP) engaged in self-assembly of ribosomes.

**FIGURE 7:**
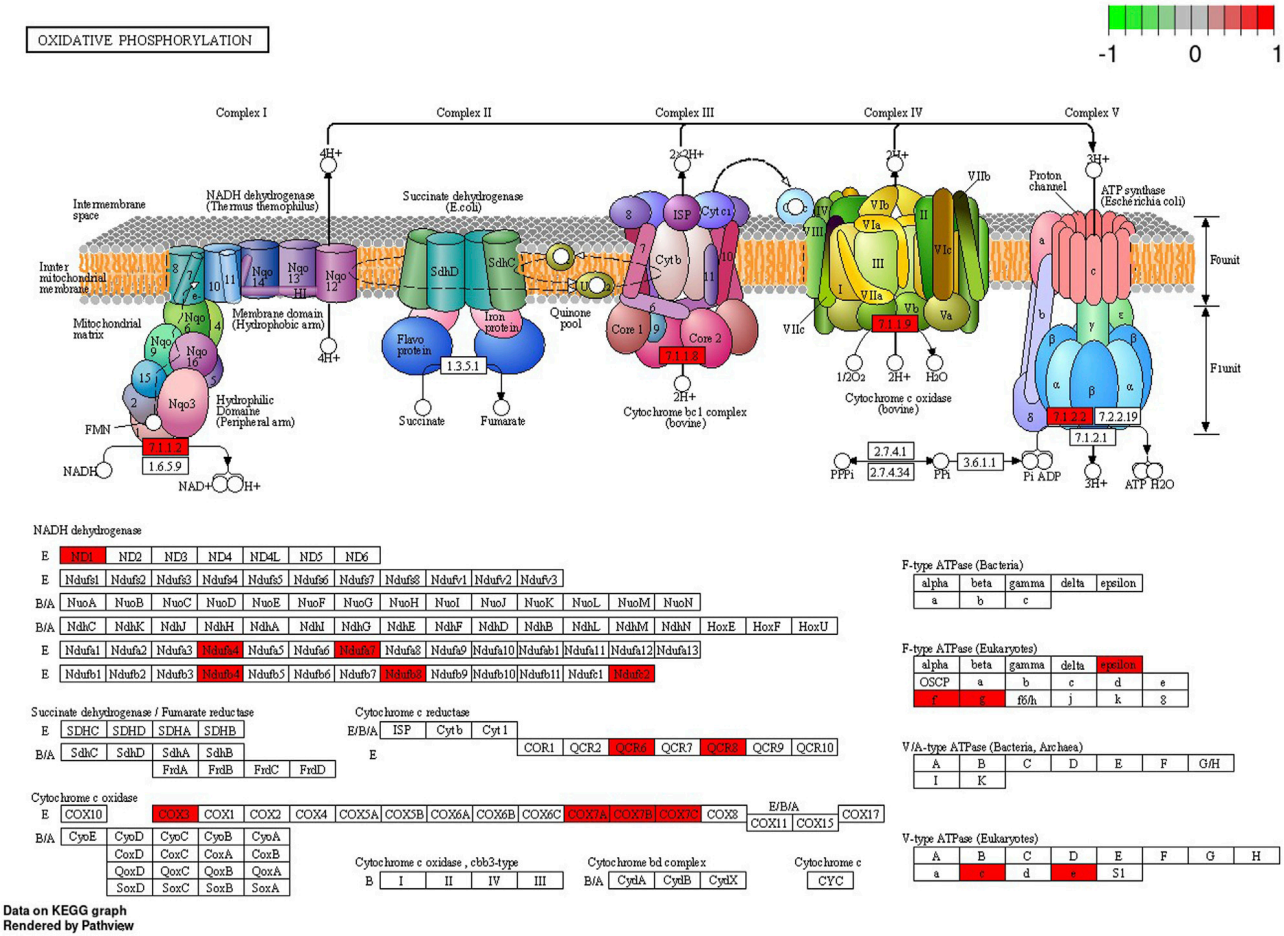
Enrichment Kyoto Encyclopedia of Genes and Genomes (KEGG) analysis of differentially expressed genes (DEGs) engaged in “Oxidative phosphorylation” signaling pathway induced in the kidney after BPA exposure. Red rectangles represent upregulated genes. Logarithmic fold change (log2FC) red-green scale describes gene expression values.

The 301 DASs-biased genes were assigned to functional GO annotations grouped into 39 BP terms, 20 MF and 16 CC categories (Supplementary Table S7, Supplementary Figure S5). Some of the DASs were also entangled in the most important KEGG pathways revealed in the BPA-treated samples (Supplementary Figure S6). Within DASs genes, there were transcripts linked directly with the function and structure of the kidney. One discovered DAS gene (*DVL1*) was a component of the Wnt signaling in kidney disease. Changes in expression of alternative splicing profiles of *DVL1* and others (*INPP5E*, *NDUFAF3*, *ARVCF*, *NEK8*, *FANCE*, *BUD23*, *MCFD2*, *MKS1*, *METTL27*, *SON*, *FANCL*, *KAT6A*, and *DNA2*) according to GO-derived data cause the potential “abnormality of the kidney”. The *HSF1* and *SIRT2* take part in the response to oxidative stress. The other interesting alternatively spliced genes were engaged in chemical carcinogenesis (*ARAF*), chronic kidney disease (*NEK8*) and kidney development (*CNTRL*, *TET2*, *NLE1*).

### 3.5 Validation of the results

The genes for validation were selected following the assessment of the expression values and read’s distribution within samples. Statistical analysis using the Pfaffl method proved the significant changes in the expression levels of 13 genes compared with the control (Supplementary Figure S7). The results showed that six genes (*ERC1*, *APOA1*, *S100A8*, *HAMP2*, *SULT2A1* and *SULT2A2*) were upregulated and seven (*PEG3*, *AKT3*, *COL19A1*, *GPLD1*, *CEP350*, *PEAK1*, and *ATM*) were downregulated. The expression profiles of all validated genes determined by Real-time PCR were similar to those obtained in the sequencing experiment. The analyzed results revealed that 7 of the validated DEGs had 14 (PEG3), 6 (ERC1), 2 (CEP350, HAMP2, SULT2A1) or 1 (ATM, S100A8) positive correlations with DELs expression (Supplementary Figure S8).

## 4 Discussion

The existing data indicate that BPA should be considered as a factor capable of inducing distinct effects on kidneys. Although it has been known, that its high concentration in the blood increases the risk of kidney damage (Hu et al., 2016), the precise molecular pathomechanisms exerted by BPA on the kidney have not been fully discovered. The results of our study revealed that BPA induces a significant impact on kidneys at the transcriptomic level. The gene expression profiling of organs taken from the control and BPA-treated mice revealed 433 DEGs (including 201 downregulated and 232 upregulated), and profound changes have been observed in several essential processes such as mitochondrial and ribosome function, oxidative phosphorylation and chemical carcinogenesis induced by reactive oxygen species.

Furthermore, this project examined also the relationship between several genes within the identified pathways and genes with alternative splicing bias, exposed by DASs, for instance within genes assigned to abnormalities of the kidney, such as Inositol Polyphosphate-5-Phosphatase E (*INPP5E*), Dishevelled Segment Polarity Protein 1 (*DVL1*), ARVCF Delta Catenin Family Member (*ARVCF*), NIMA Related Kinase 8 (*NEK8*) or SON DNA and RNA Binding Protein (*SON*) (Walter et al., 2009; Zalli et al., 2012; Hakim et al., 2016; Sharifian et al., 2018; Kim et al., 2019).

It has been found, that BPA excreted by the kidneys can contribute to progressive, cumulative kidney damage throughout life, caused by oxidative stress and mitochondrial dysfunction (Kobroob et al., 2018). Kidneys are highly susceptible to reactive oxygen species (ROS) damage due to the abundance of long-chain polyunsaturated fatty acids in renal lipids. It has been observed, that 5 weeks long exposure to a low dose (50 mg/kg) of BPA in rats leads to apparent renal dysfunction manifested by glomeruli impairment, mitochondrial swelling, and increased ROS production. Moreover, the high dose of BPA (150 mg/kg) causes sclerosis of the glomeruli, atrophy, and damage to the basal membrane, and many of these pathological changes are caused via oxidative stress mechanisms (Kobroob et al., 2018). The results of the present study confirm previous findings, as many of the molecular changes induced by BPA in renal tissue were associated with the mitochondrial oxidative phosphorylation (OXPHOS) process, implying that the imbalance between the production and accumulation of ROS is one of the main mechanisms induced by BPA in kidneys. In the BPA-treated samples, altered gene expression has been identified in “Oxidative phosphorylation” KEGG pathway. Additionally, the essential genes were annotated to ontological terms within BP ontological terms, such as: “Oxidative phosphorylation”, “Mitochondrial ATP synthesis coupled electron transport”, “Mitochondrial ATP synthesis coupled electron transport”, “Aerobic respiration”, “Aerobic electron transport chain”, “Mitochondrial respiratory chain complex assembly” or “Mitochondrion organization”. Further, the present data revealed two alternatively spliced genes, namely Sirtuin 2 (*SIRT2*) and Heat Shock Transcription Factor 1 (*HSF1*) entangled in response to oxidative stress, encoding factors that are activated for instance in kidney damage (Lou et al., 2019; Ogura et al., 2021).

Since OXPHOS generates ATP for mammalian cells, it is not surprising that an inadequate mitochondrial energy supply can cause deleterious dysfunctions in organs that require a great deal of energy (Bhargava and Schnellmann, 2017). The kidneys demand a significant number of efficient mitochondria to fulfil their functions, i.e. to eliminate waste products and to control fluid and electrolyte balance. Consequently, renal mitochondrial impairment results in disturbances in ATP production and thus influences cellular structure (Bhargava and Schnellmann, 2017). OXPHOS consists of five multi-subunit enzymes called complexes I - V. Electrons are transferred across complexes I - IV in conjunction with proton transfer through the inner membrane. By converting electrochemical potential into ATP by H+-ATP synthase, it is translated into chemical energy (Kontro et al., 2015). The results of the present study revealed that BPA administration was followed by upregulated expression of genes associated with complexes I, III, IV and V of the “Oxidative phosphorylation” pathway. Most of the DEGs associated with this pathway are engaged in the coding of subunits of complex I (NADH: ubiquinone oxidoreductase) and complex IV - cytochrome c oxidase (COX). Considering genes related to subunits of complex I, the present study uncovered upregulation of five genes, namely: NADH-Ubiquinone Oxidoreductase MLRQ Subunit (*NDUFA4*), Ubiquinone Oxidoreductase Subunit C (*NDUFC2*), Ubiquinone Oxidoreductase Subunit A7 (*NDUFA7*), Ubiquinone Oxidoreductase Subunit B4 (*NDUFB4*) and Ubiquinone Oxidoreductase Subunit B8 (*NDUFB8*). Complex I, located in the inner mitochondrial membrane, is crucial for respiration in many aerobic organisms and controls oxidative phosphorylation and mitochondrial respiration (De Paepe et al., 2012). Among its function, it oxidizes NADH from the tricarboxylic acid cycle, assists in the reduction of ubiquinone, and transports protons across the inner membrane. In addition, it is a major contributor to the production of ROS within cells (Vinothkumar et al., 2014). Our research revealed also the upregulation of S100 Calcium Binding Protein A8 (*S100A8*) encoding a molecule that is mainly found in calprotectin form (S100A8/S100A). The overexpression of S100A8 leads to mitochondrial complex I inhibition and causes mitochondrial dysfunction (Li et al., 2019). Moreover, the present study revealed, that several genes upregulated in the BPA-treated kidney samples, namely: Mitochondrially Encoded Cytochrome C Oxidase III (*COX3* also known *MT-CO3*), Cytochrome C Oxidase Subunit 7A (*COX7A*), Cytochrome C Oxidase Subunit 7B (*COX7B*) and Cytochrome C Oxidase Subunit 7C (*COX7C*), were associated with complex IV of OXPHOS. COX is a component of the mitochondrial respiratory chain, responsible for transferring electrons from reduced cytochrome c to molecular oxygen. It has been found, that COX deficiency leads to severe mitochondrial disorders (Kogot-Levin et al., 2016). Researchers observed that decreased COX activity in human fibroblasts results in compromised ATP synthesis, reactive oxygen species overproduction, and abnormal mitochondrial morphology (De Paepe et al., 2012). Additionally, COX dysfunctions and age-related inactivity have been reported in Alzheimer’s and Parkinson’s diseases (Kogot-Levin et al., 2016).

Interestingly, the overexpressed genes encoding subunits of complex I, III-V of the OXPHOS have been assigned also to other KEGG pathways identified in the BPA-treated kidneys, including “Chemical carcinogenesis - reactive oxygen species”. Therefore, the present results confirm the available data suggesting that BPA exposure may increase the risk of cancer incidence (Brandt et al., 2014). Moreover, it has been revealed that BPA can also induce resistance in many types of cancer cells to chemotherapeutics such as doxorubicin, cisplatin, carboplatin and tamoxifen (Hafezi and Abdel-Rahman, 2019). The present study revealed in BPA-treated samples several DEGs which potentially may contribute to the carcinogenic potential of BPA, for instance, the underexpression of Paternally Expressed 3 (*PEG3*). It has been previously found, that the *PEG3* expression is significantly reduced in renal clear cell carcinoma (ccRCC) compared to non-tumor renal tissue, and *in vitro* studies have shown that knockout of *PEG3* causes acceleration of ccRCC proliferation. Findings suggest that *PEG3* is indispensable for the regulation of ccRCC progression (Qiu et al., 2023). Another DEG associated with cancer revealed in BPA-treated tissues is Apolipoprotein A1 (*APOA1*). APOA1 plays a crucial part in lipid metabolism, however, by regulating cholesterol export and dampening *COX-2* expression, *APOA1* overexpression could curb the malignancy of cancer (Zeng et al., 2022). Furthermore, our research has shown underexpression of genes associated with tumor formation and cancer cell migration, including AKT Serine/Threonine Kinase 3 (*AKT3*), Serine/Threonine Kinase (*ATM*) and Pseudopodium Enriched Atypical Kinase 1 (*PEAK1*) gene (Bristow et al., 2013; Weber and Ryan, 2015; Grottke et al., 2016).

Considering our results, the carcinogenic effect of BPA is exerted mainly by the disturbances in OXPHOS and ROS production processes, not surprisingly, as accumulating ROS is reported to play a crucial role in signal transduction, cell differentiation and proliferation, leading to activation of oncogenic pathways (Hafezi and Abdel-Rahman, 2019). It is also well known that oxidative stress causes deleterious modifications of DNA (including gene mutations and altered gene expression) leading to tumor development (Franco et al., 2008). It should be also mentioned, that BPA administration was followed by overexpression of Lactoperoxidase (*LPO*), assigned to the “Chemical carcinogenesis - reactive oxygen species” pathway. This gene encodes a member of the peroxidase family of proteins which participate in the generation of the antimicrobial substance hypothiocyanous acid (Nichol et al., 1987). However, it has been also revealed that *LPO* has mutagenic and carcinogenic action through its capacity to generate free radicals (Jahanbakhsh et al., 2020). Moreover, it has been found that LPO is engaged in breast cancer etiology due to its ability to activate heterocyclic amines belonging to environmental and dietary carcinogens (Sheikh et al., 2017). Thus the upregulation of *LPO*, together with OXPHOS imbalance, observed after BPA-treatment, may contribute to the increased risk of kidney cancer development. Furthermore, the “Chemical carcinogenesis - reactive oxygen species” pathway was also exposed by alternatively spliced A-Raf Proto-Oncogene, Serine/Threonine Kinase (*ARAF*, also known as *RAF*). This gene encodes Raf family kinases involved in the Ras-Raf-MAPK pathway participating in cell cycle regulation, proliferation and differentiation, survival and apoptosis. *ARAF* alteration is a common indicator in cancer and contributes to tumor initiation, progression and metastasis (Leicht et al., 2007). Furthermore, ARAF is altered in renal cell carcinoma patients (AACR Project GENIE Consortium, 2017).

Surprisingly, although the present study focused on the influence of BPA on renal tissue, bioinformatics resources disclosed a collection of DEGs annotated in two KEGG pathways “Alzheimer’s disease” and “Parkinson’s disease”. Of course, we are aware of the fact that these two diseases affect neurons within the brain. However, it cannot be excluded that similar KEGG signaling interactions may be triggered in the central nervous system, especially since the interconnection between kidney impairment and increased risk of the above-mentioned mental diseases has been previously discovered for instance in patients suffering from Chronic Kidney Disease (CKD) (Zhang et al., 2020). The main cause of CKD is a bad lifestyle which leads to obesity, diabetes and hypertension. Moreover, increased blood levels of BPA have been observed in patients suffering from this disease (Krieter et al., 2013). The observation of altered gene expression in KEGG pathways associated with both Alzheimer’s and Parkinson’s in BPA samples is highly alarming. Considering that most energy comes from oxidative phosphorylation, brain tissue is highly susceptible to ROS. It has been revealed that BPA affects the blood-brain barrier, and its increased plasma levels exert neurotoxicity and neuroinflammation by enhancing oxidative stress (Engin and Engin, 2021). Moreover, this effect may be accelerated by the fact that BPA, by impairing glomerular filtration, may lead to the decreased renal elimination of this substance and thus further increase in blood concentration. Due to the present findings, the main pathomechanism exerted by BPA associated with these two diseases is again connected with disturbances in OXPHOS.

In BPA-treated kidneys, the present study revealed modified DEGs enriched in the “Ribosome” KEGG pathway. Furthermore, several altered genes associated with ribosome function were annotated to ontological terms within processes including MF (“Structural Constituent of Ribosome”), BP (“Ribosomal Small Subunit Biogenesis”, “Ribosomal Small Subunit Assembly”, “Ribosome assembly”, “Ribosome Biogenesis”) and CC (“Cytosolic large ribosomal subunit”, “Large Ribosomal Subunit”). Ribosomal proteins (RP) are the main components of ribosomes and are essential for proper cell growth and maintenance (Dai and Wei, 2017). Our research revealed upregulation of several genes coding RP related to large subunit (*RPL*), such as *RPLP1*, *RPL9*, *RPL11*, *RPL12*, *RPL13A*, *RPL17*, *RPL22*, *RPL31*, *RPL32*, *RPL34*, *RPL35*, *RPL35A*, *RPL36*, *RPL36A*, *RPL36A-PS1*, *RPL39*, and *RPL41*. A variety of physiological and pathological cellular activities, including protein synthesis, cell proliferation, DNA repair and tumorigenesis are regulated by RP (Fan et al., 2017). Thus the altered expression of these genes may again lead to the impairment of kidney function and enhanced carcinogenesis.

## 5 Study limitations and future work

The main goal of this study was to perform an analysis of the kidney transcriptome and define changes in the expression profile caused by the influence of BPA. The obtained results constitute the basis for the selection of significant genes and enriched pathways that should next be evaluated functionally, however, this remains beyond the purpose of the current research and should be determined in the future. In the current manuscript, we focused on the description of the DEGs and the co-expression analysis performed to reveal DEGs−DELs *trans*-interactions. As described in the manuscript, identified events showed the mediation of 74 DELs in the regulation of 176 DEGs. The majority of DEGs−DELs *trans*-actions were positively correlated, and only 5 showed a negative correlation of expression. Moreover, the analyzed results revealed that among the validated DEGs 7 had 14 (*PEG3*), 6 (*ERC1*), 2 (*CEP350*, *HAMP2*, *SULT2A1*) or 1 (*ATM*, *S100A8*) positive correlations with DELs expression. Such an approach has been selected as lncRNA is emerging as equally important to DEGs and it is intended to fully analyze possessed data in terms of lncRNA and present it in a separate research. During DEGs−DELs interaction analyses, *cis*-actions have been also examined based on the colocalization of genes encoding these molecules. Unfortunately, a procedure that has been used many times in our previous studies (Majewska et al., 2018; Paukszto et al., 2020; Makowczenko et al., 2022; Makowczenko et al., 2023), did not reveal any significant *cis*-interactions in the current research. 80 correlation pairs (DELs and DEGs) located in the same chromosomes have been identified and none of the DELs were localized in the vicinity (10,000 bp) of DEGs. Due to the lack of conclusive results, this section of bioinformatics analyses was omitted.

## 6 Conclusion

To the best of our knowledge, this is the first study concerning the thorough investigation of the changes in the gene expression profiling, posttranscriptional modifications and pathway analysis of kidneys after oral exposure to BPA in mice. BPA is used globally in the production of polycarbonate plastics, which is why the main exposure to BPA comes from food and water primarily due to the direct contact with food containers and other materials used in the course of production, handling, and transportation. BPA can disturb the homeostasis of the human endocrine system, resulting in reproductive and developmental dysfunctions. Moreover, BPA enhances cancer development, triggers obesity, and causes respiratory tract disorders. Which is a serious problem, is that the obtained results indicate that BPA exposure causes profound changes in several critical processes, including oxidative phosphorylation, mitochondrial and ribosome function, and chemical carcinogenesis induced by reactive oxygen species. Moreover, evidence from this study indicates the altered gene expression in functional pathways associated with both Alzheimer’s and Parkinson’s diseases. The transcriptomic findings of this research shed light on how BPA affects kidney function and broadens the potential target points for clinical interventions. Therefore, we hope that this research lays the groundwork for studies and clinical trials regarding the possible effect of BPA on other organ dysfunctions.

## Data Availability

The data presented in the study are deposited in the European Nucleotide Archive repository, accession number PRJEB59035 (https://www.ebi.ac.uk/ena/browser/view/PRJEB59035).
