# Supplementary material for: A comprehensive transcriptomic analysis of the bisphenol A affected kidney in mice
Source: Front Mol Biosci. 2023 Nov 24;10:1260716. doi: 10.3389/fmolb.2023.1260716 (PMC10704486; doi:10.3389/fmolb.2023.1260716)
Supplement: Supplementary file 2 [file Table1.DOCX]

Table S1: The list of primers used for Real-time PCR.

| Name | Forward sequence: (5' to 3') | Reverse sequence: (5' to 3') |
| --- | --- | --- |
| *PEG3* | ATCAAGGATGTGGCTCGCAA | GCTTTTATGCTCATGGCCCG |
| *AKT3* | ACCGCACACGTTTCTATGGT | TGACAACACCTAAGCCCCAC |
| *COL19A1* | AGAGGAGAACCCGGCCTATT | GGTTTCCCTGGCAGGCTTAT |
| *ERC1* | GCGGACACCACTAACACAGA | GGAAGCCAGGGAAGAAGCAT |
| *GPLD1* | GGACTGCTAGGGGGAGAAGA | CTTCCAGCAGCAACAACCAC |
| *APOA1* | GCACGTATGGCAGCAAGATG | CCGTTCCTGCAGCTGACTAA |
| *CEP350* | TCCGAAAGTGATGCTGCTGT | GTGCTGTTGCCACTTTCCTG |
| *S100A8* | TGCCGTCTGAACTGGAGAAG | GTGAGATGCCACACCCACTT |
| *HAMP2* | CTCCTGCTTCTCCTCCTTGC | ACCACAGGAGGGTTTGTTACA |
| *PEAK1* | CAAAACGGAAGGGGCACAAG | TGGCAGTAGGGCTTTCTGTG |
| *SULT2A1* | CCAAGGGAGATCCGAAGTGG | AGTTCCGAGTGACCCTGGAT |
| *SULT2A2* | CCAAGGGAGATCCGAAGTGG | TCCGAGTGACCCTGGATTCT |
| *ATM* | GATCTGCTCATTTGCTGCCG | CTGTCTGGAGCTCTGTGTGG |
| *UBC* | CCCAGTGTTACCACCAAG | ATCACACCCAAGAACAAGC |
| *ACTB* | GCCAACCGTGAAAAGAT | AGAGCATAGCCCTCGTAGAT |
