# Supplementary figures and images for: A comprehensive transcriptomic analysis of the bisphenol A affected kidney in mice
Source: Front Mol Biosci. 2023 Nov 24;10:1260716. doi: 10.3389/fmolb.2023.1260716 (PMC10704486; doi:10.3389/fmolb.2023.1260716)

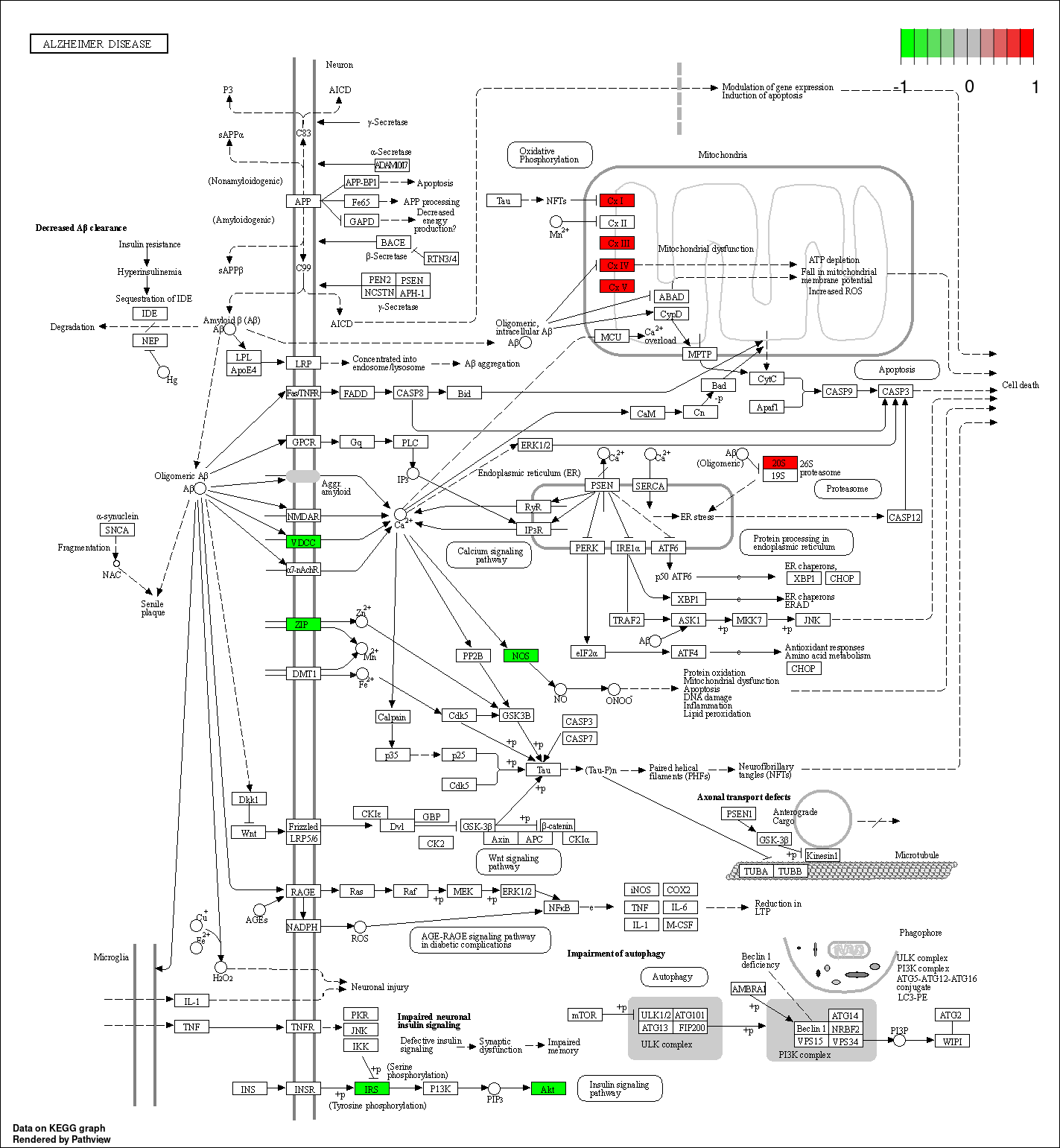

Supplement: Supplementary file 3 [file Image5.PNG]

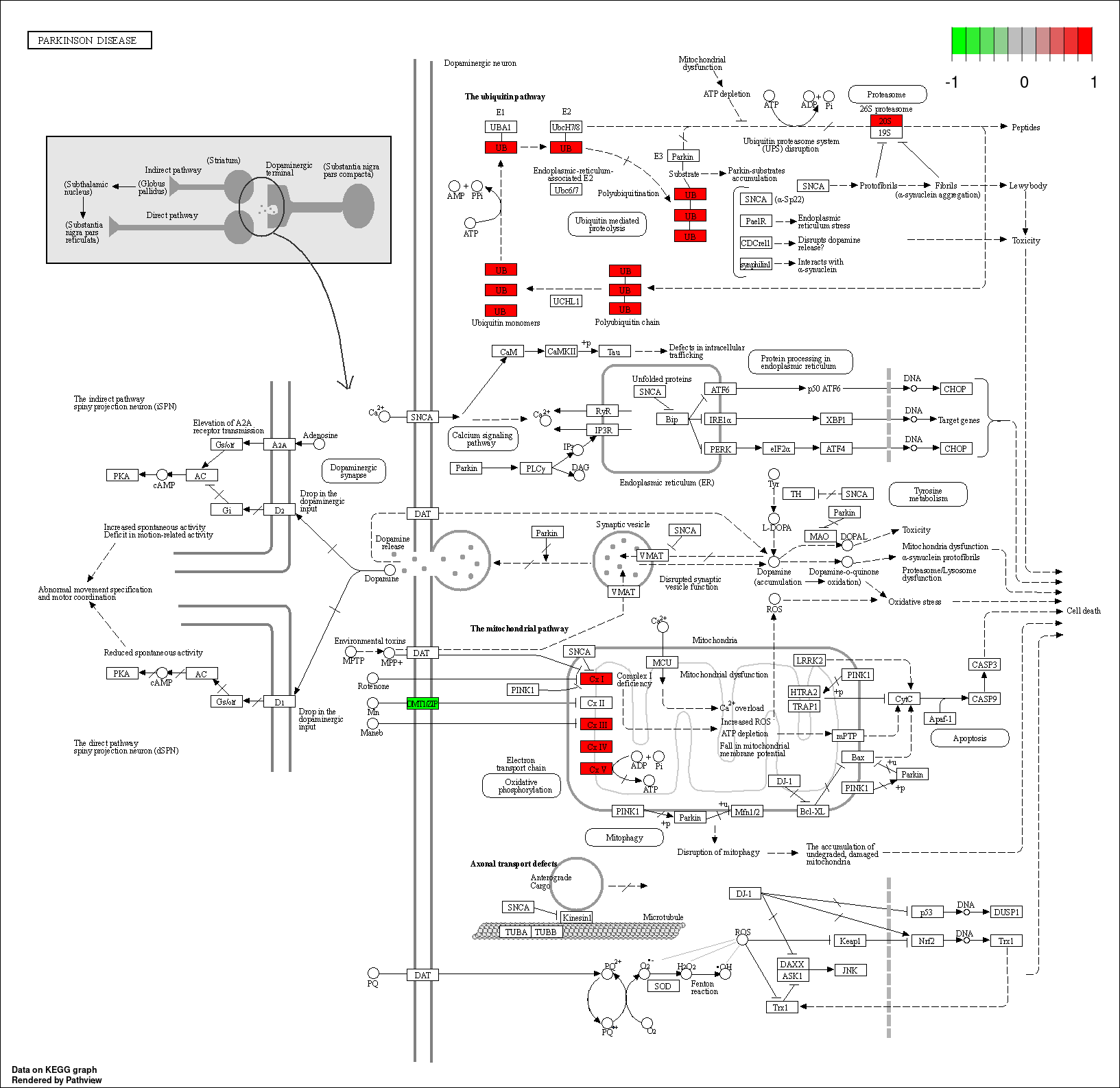

Supplement: Supplementary file 4 [file Image4.PNG]

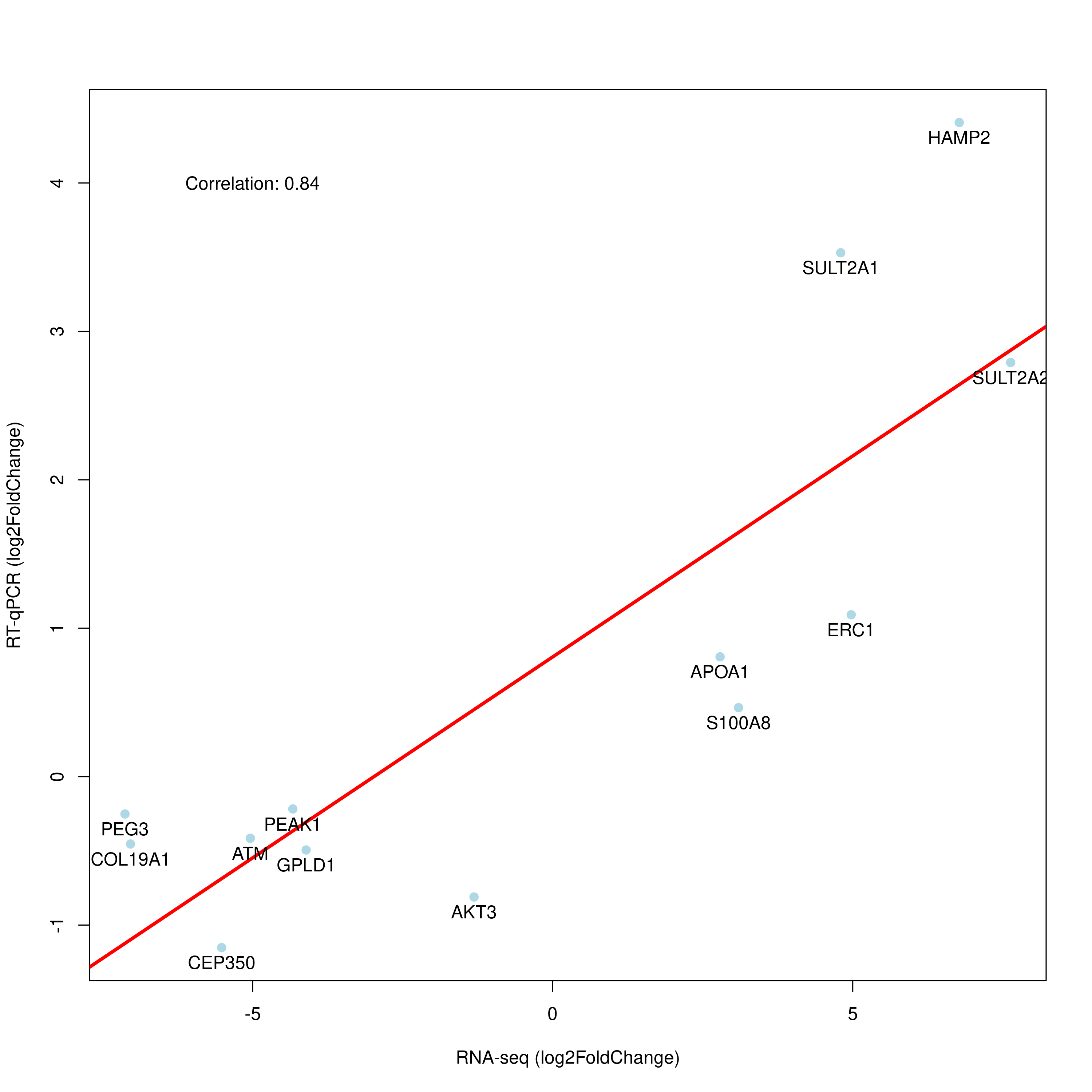

Supplement: Supplementary file 10 [file Image2.PNG]

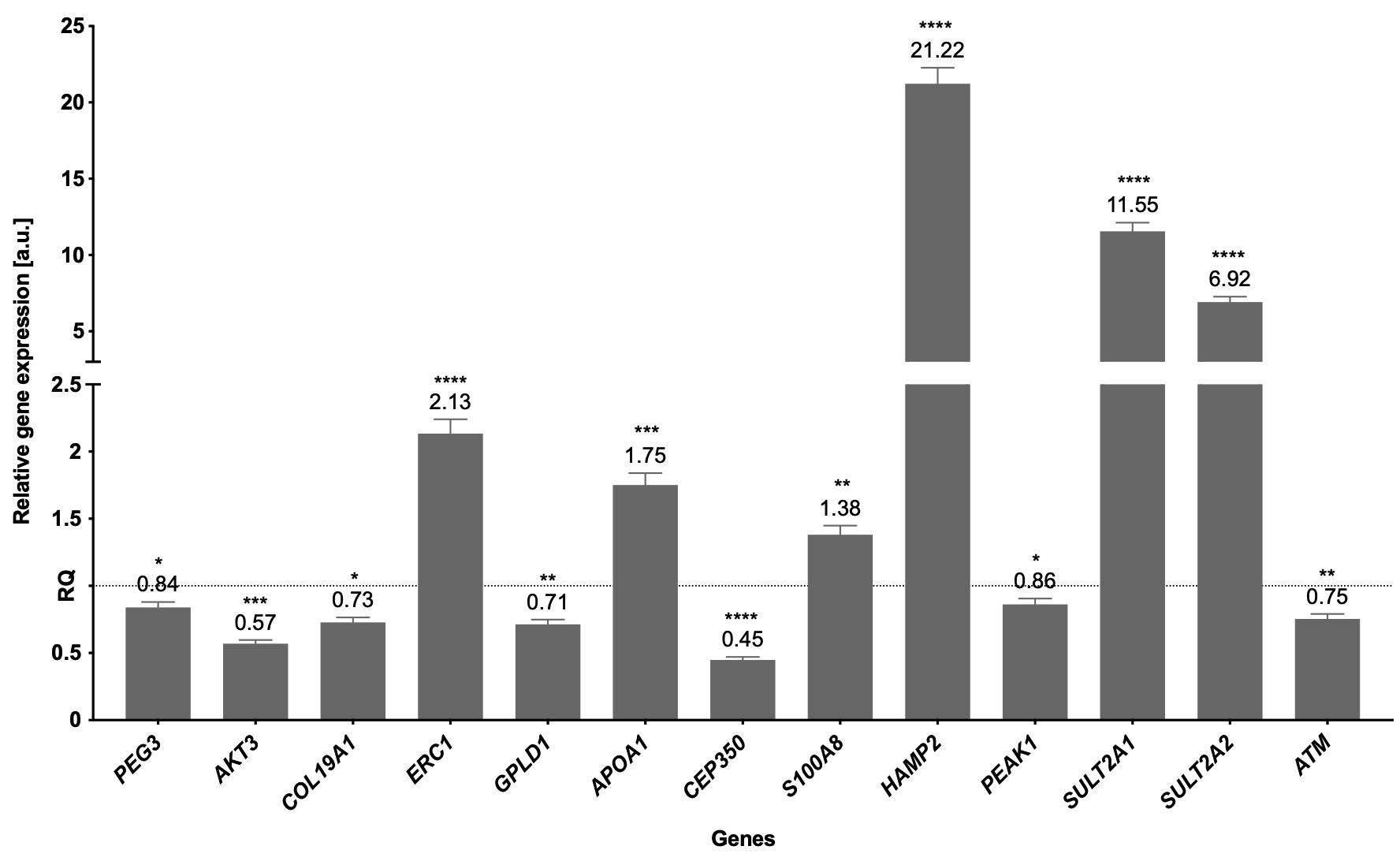

Supplement: Supplementary file 11 [file Image1.PNG]

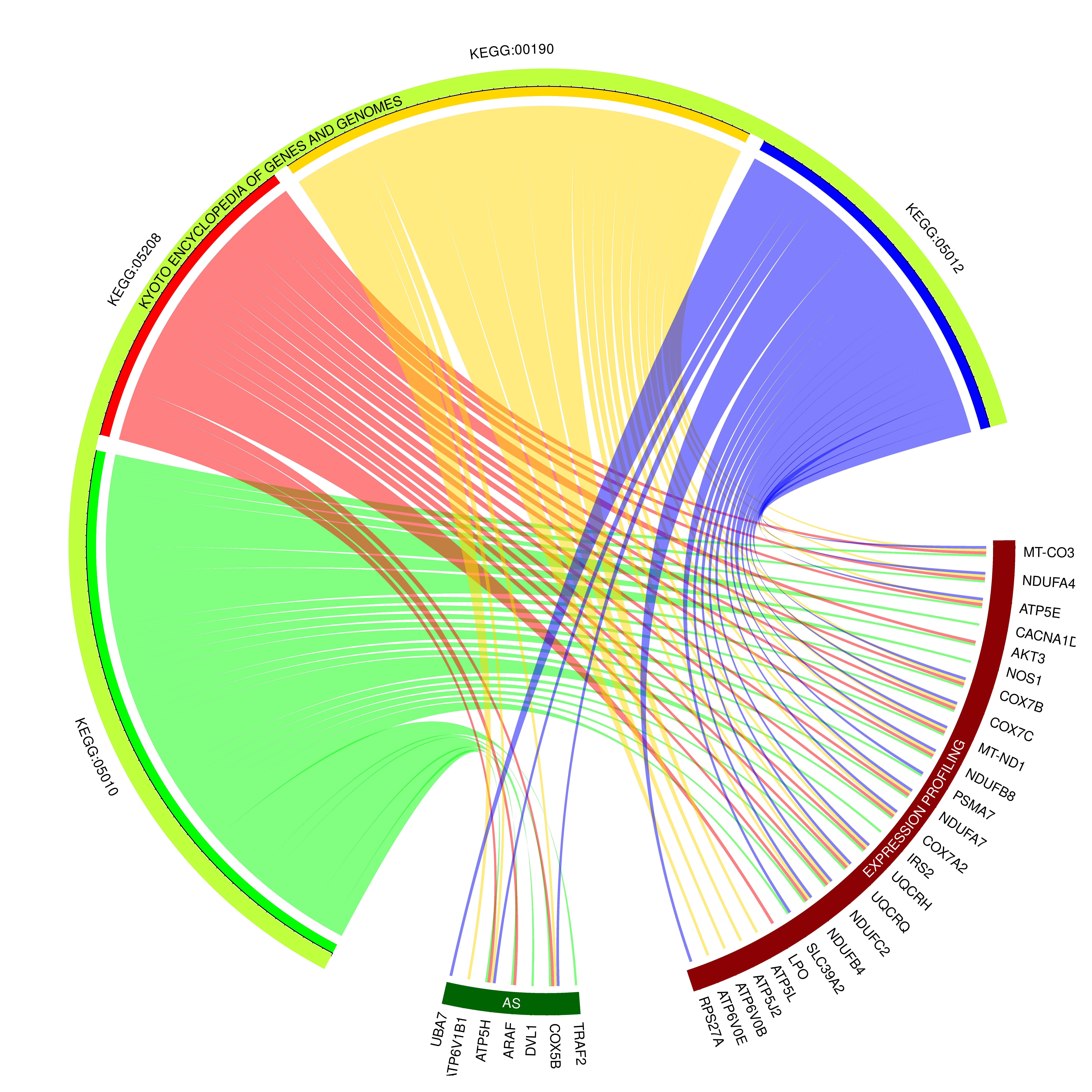

Supplement: Supplementary file 12 [file Image8.PNG]

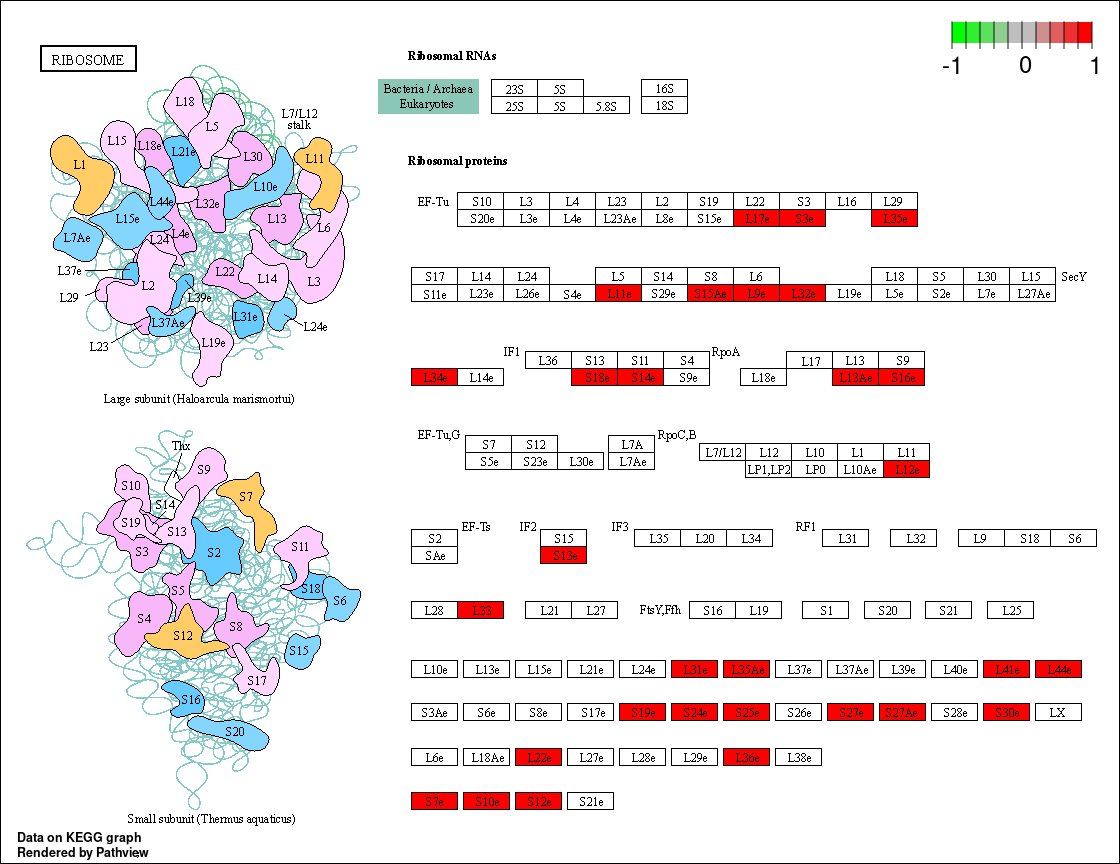

Supplement: Supplementary file 14 [file Image6.PNG]

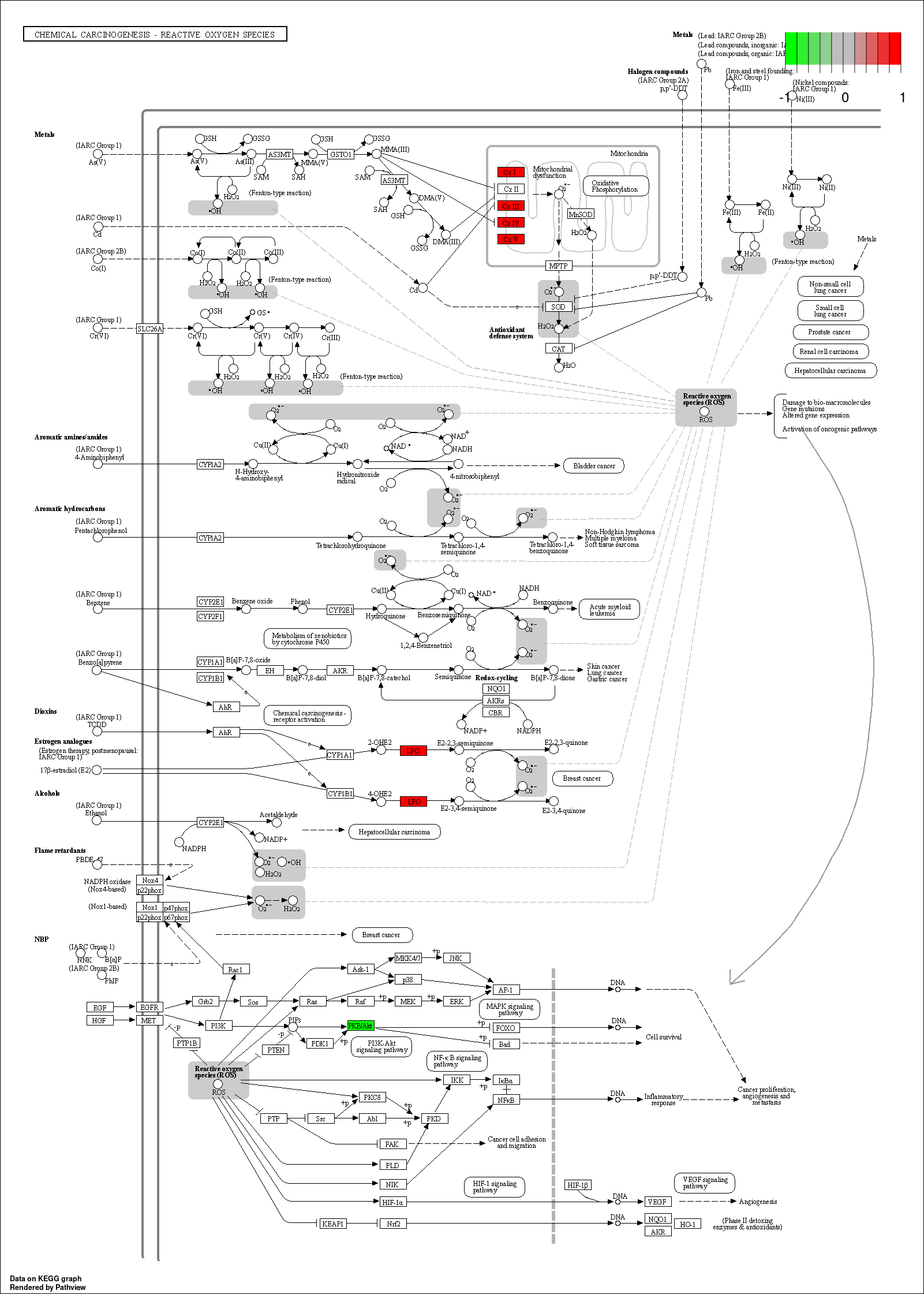

Supplement: Supplementary file 16 [file Image3.PNG]
