# Supplementary material for: A comprehensive transcriptomic analysis of the bisphenol A affected kidney in mice
Source: Front Mol Biosci. 2023 Nov 24;10:1260716. doi: 10.3389/fmolb.2023.1260716 (PMC10704486; doi:10.3389/fmolb.2023.1260716)
Supplement: Supplementary file 6 [file Table2.DOCX]

**Supplementary Table 2** The statistical metrics for the RNA libraries.

| Reads | **CTR1** | **CTR2** | **CTR3** | **CTR4** | **CTR5** | **CTR6** | **BPA1** | **BPA2** | **BPA3** | **BPA4** | **BPA5** | **BPA6** |
| --- | --- | --- | --- | --- | --- | --- | --- | --- | --- | --- | --- | --- |
| Row | 61.69 | 63.07 | 62.81 | 60.91 | 62.77 | 63.41 | 65.38 | 63.65 | 64.03 | 61.58 | 62.55 | 61.48 |
| Trimmed | 49.63 | 53.72 | 53.39 | 52.17 | 55.47 | 53.34 | 55.26 | 53.66 | 53.73 | 53.00 | 51.94 | 53.09 |
| Mapped | 47.70 | 47.93 | 47.97 | 49.65 | 53.06 | 50.18 | 53.57 | 51.76 | 52.24 | 50.81 | 50.26 | 49.24 |
| Uniquely mapped | 41.87 | 42.18 | 42.47 | 43.86 | 46.98 | 44.69 | 46.30 | 45.20 | 45.08 | 43.52 | 43.95 | 43.04 |
| Uniquely mapped | 87.77% | 88.01% | 88.54% | 88.35% | 88.55% | 89.07% | 86.42% | 87.32% | 86.29% | 85.65% | 87.44% | 87.40% |
| Multi-mapped | 5.83 | 5.75 | 5.50 | 5.79 | 6.08 | 5.48 | 7.28 | 6.56 | 7.16 | 7.29 | 6.31 | 6.20 |

The sequencing and mapping results for the 12 RNA-seq libraries: CTR (1–6) refers to the controls; BPA (1–6) refers to kidneys affected by BPA. The uniquely-mapped values were related to the reads mapped to only one location of the Mus musculus genome. The multi-mapped values were assigned to reads aligned to more than one locus on the reference genome. The raw, trimmed and mapped values denote in millions.
