# Supplementary material for: A comprehensive transcriptomic analysis of the bisphenol A affected kidney in mice
Source: Front Mol Biosci. 2023 Nov 24;10:1260716. doi: 10.3389/fmolb.2023.1260716 (PMC10704486; doi:10.3389/fmolb.2023.1260716)
Supplement: Supplementary file 7 [file DataSheet1.PDF]

Supplementary Figures

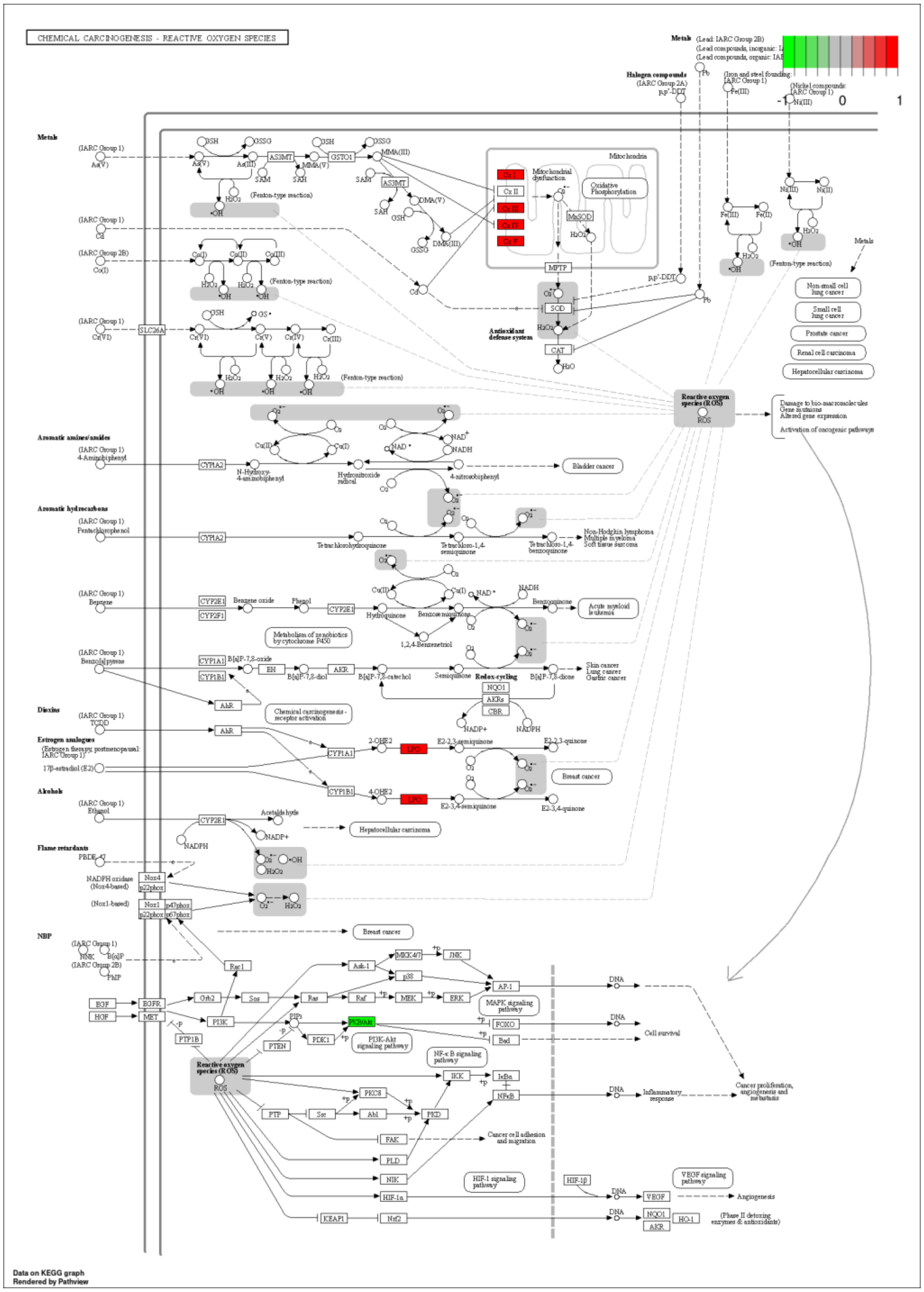

Supplementary Figure 1. Enrichment Kyoto Encyclopedia of Genes and Genomes (KEGG) analysis of differentially expressed genes (DEGs) engaged in “Chemical carcinogenesis - reactive oxygen species” signaling pathway induced in the kidney after BPA exposure. Green and red rectangles present downregulated and upregulated genes, respectively. Logarithmic fold change (log2FC) red-green scale describes gene expression values.

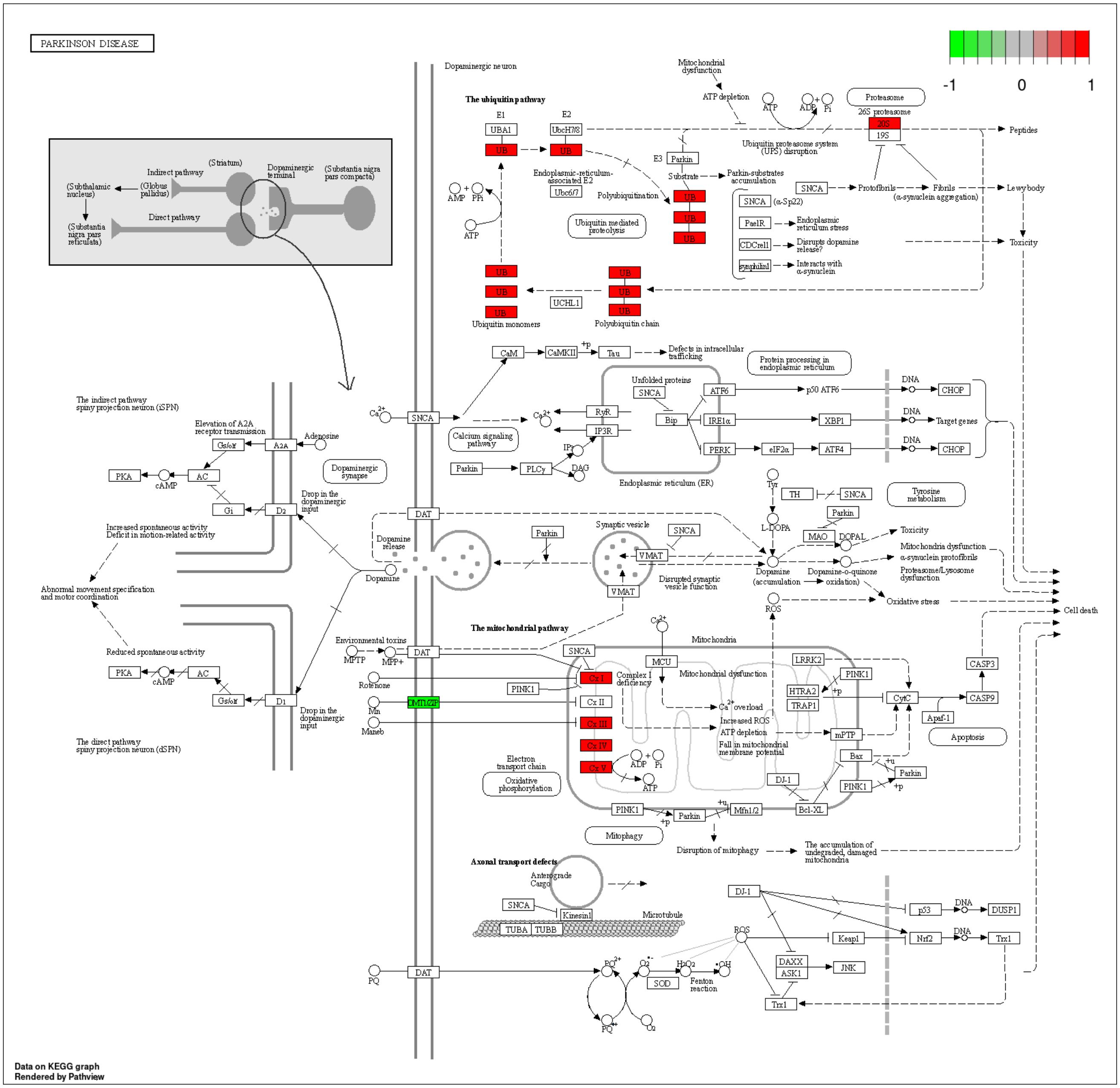

Supplementary Figure 2. Enrichment Kyoto Encyclopedia of Genes and Genomes (KEGG) analysis of differentially expressed genes (DEGs) engaged in "Parkinson disease" signaling pathway induced in the kidney after BPA exposure. Green and red rectangles present downregulated and upregulated genes, respectively. Logarithmic fold change (log2FC) red-green scale describes gene expression values.

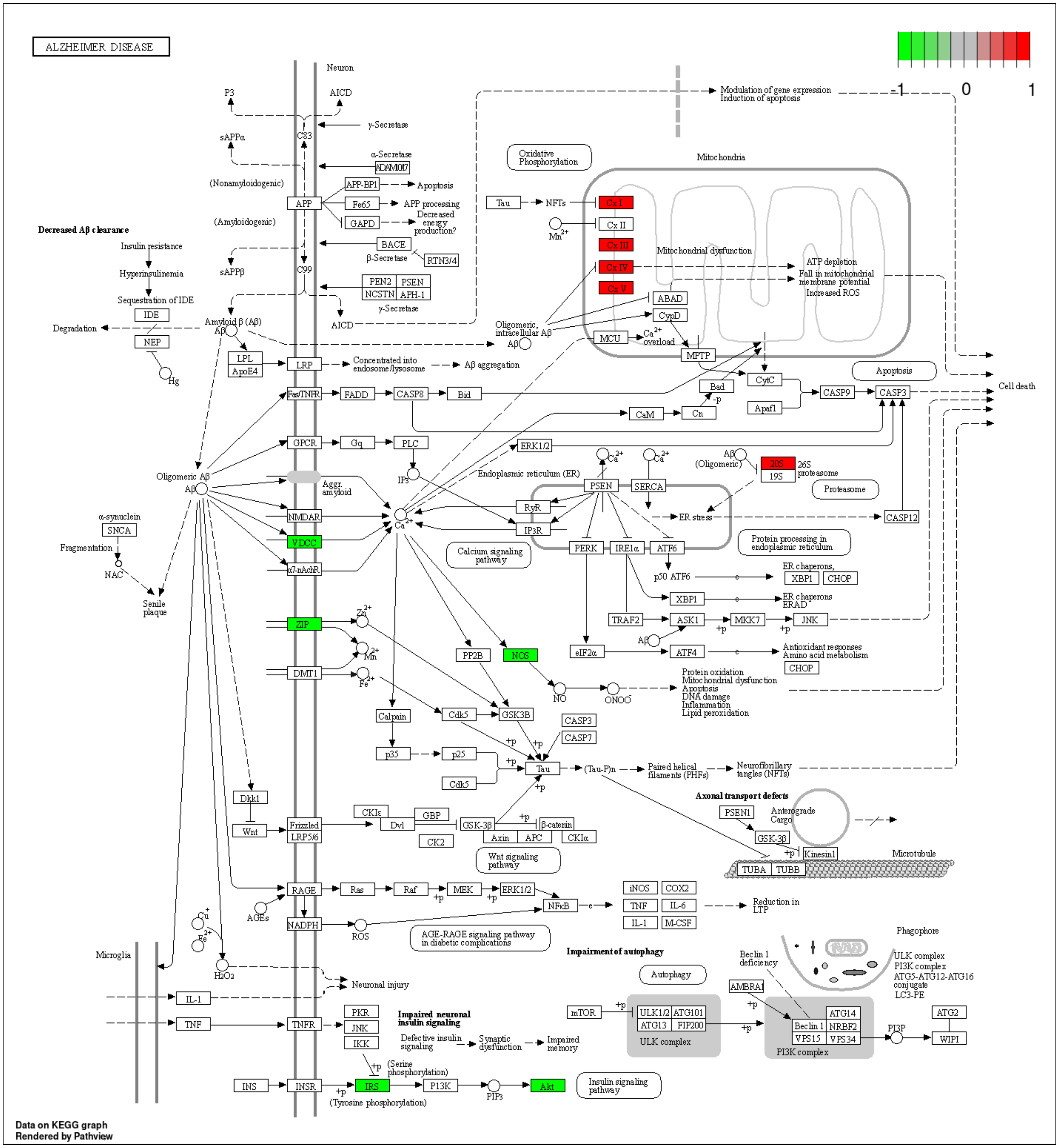

Supplementary Figure 3. Enrichment Kyoto Encyclopedia of Genes and Genomes (KEGG) analysis of differentially expressed genes (DEGs) engaged in "Alzheimer's disease" signaling pathway induced in the kidney after BPA exposure. Green and red rectangles present downregulated and upregulated genes, respectively. Logarithmic fold change (log2FC) red-green scale describes gene expression values.

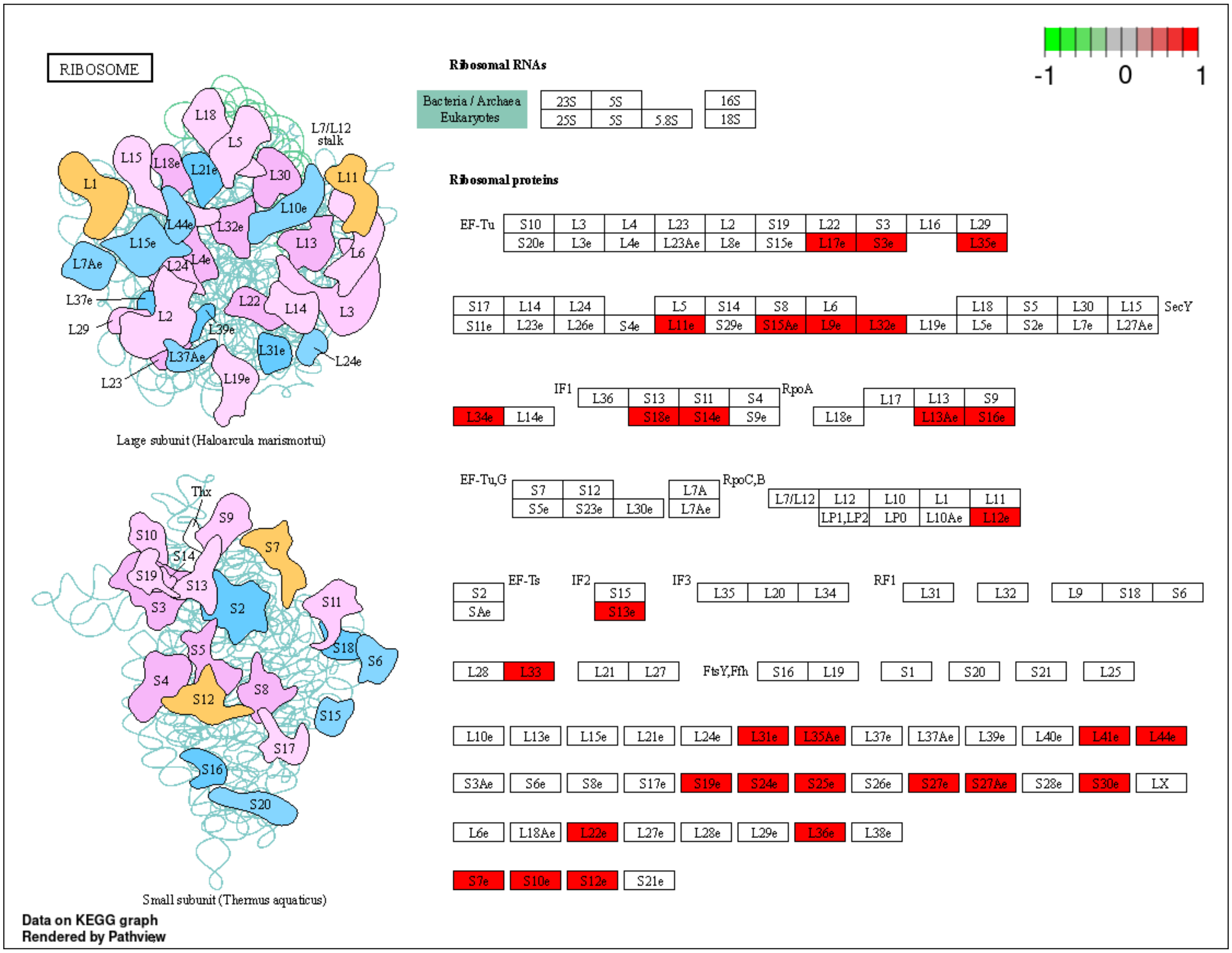

Supplementary Figure 4. Enrichment Kyoto Encyclopedia of Genes and Genomes (KEGG) analysis of differentially expressed genes (DEGs) engaged in "Ribosome" signaling pathway induced in the kidney after BPA exposure. Red rectangles present upregulated genes. Logarithmic fold change (log2FC) red-green scale describes gene expression values.

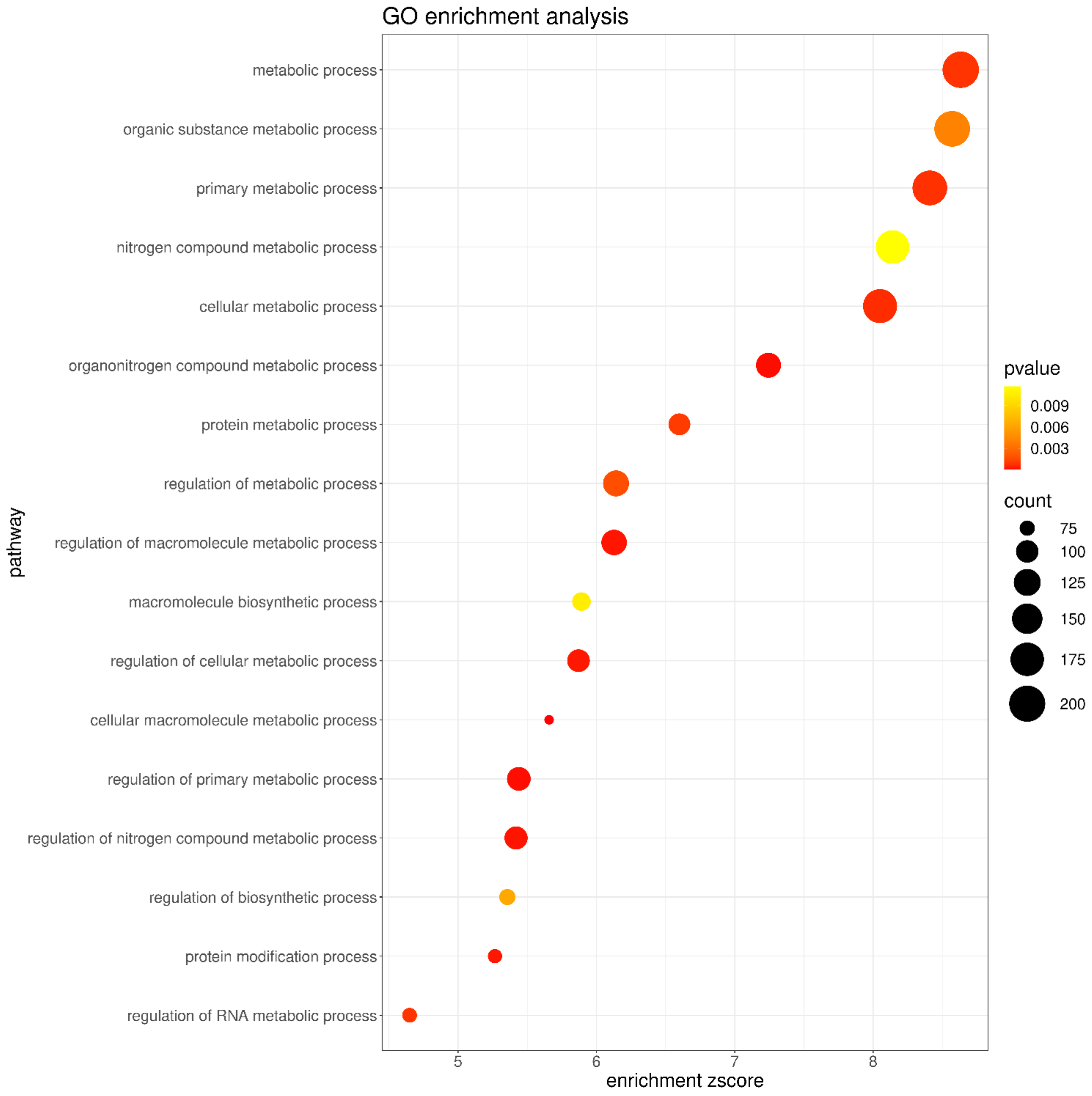

Supplementary Figure 5. Dotplot chart of enrichment ontology of DASs. The circles represent pathways described along the y-axis, colors reflect the adjusted p-value of enrichment statistics, and the sizes of the circles represent the number of genes enriched in each pathway

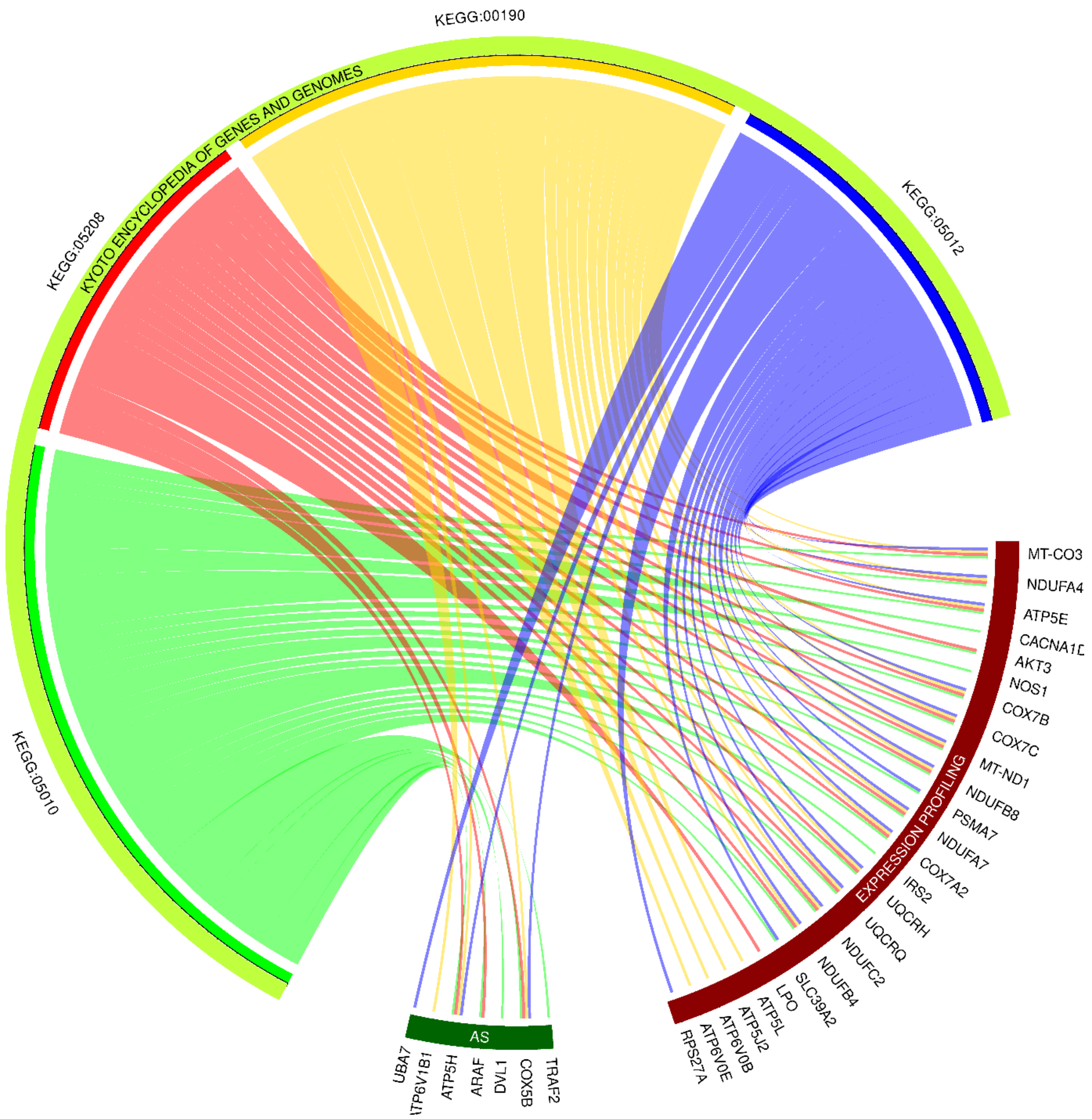

Supplementary Figure 6. Differentially expressed genes (DEGs) and alternative splicing (DASs) engaged in “Oxidative phosphorylation” KEGG signaling pathway induced in the kidney after BPA exposure. Red rectangles represent upregulated genes. Logarithmic fold change (log2FC) red-green scale describes gene expression values.

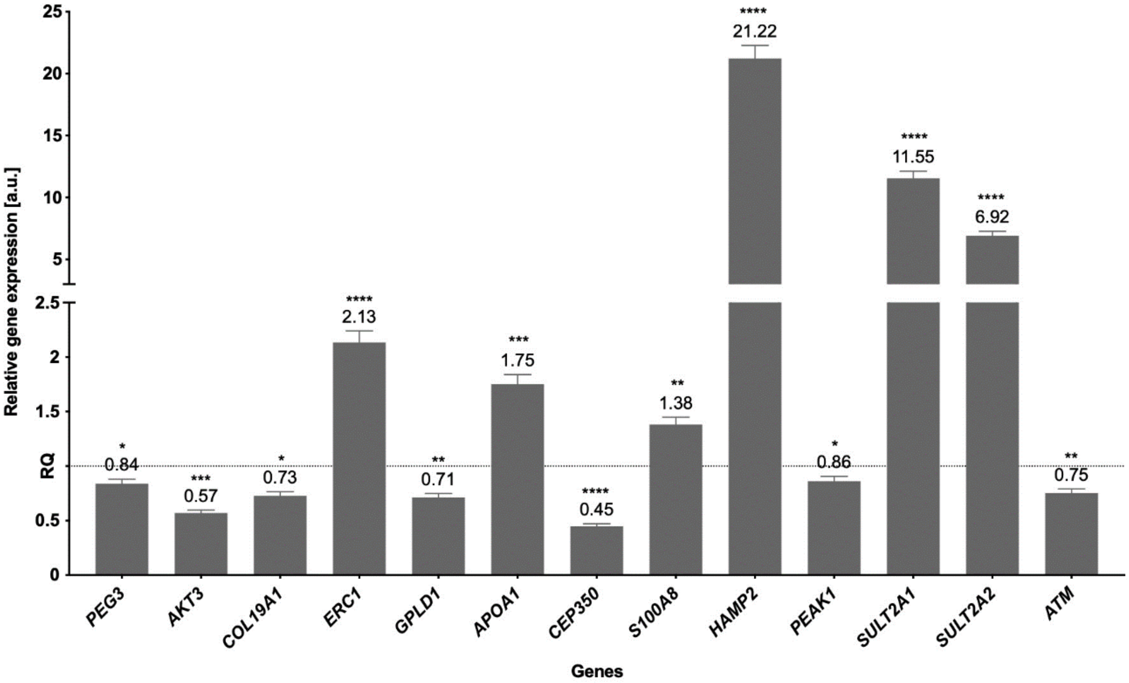

Supplementary Figure 7. The mRNA expression of selected genes obtained using Real-time PCR. The expression of endogenous control is shown as normalized to a value 1 (RQ), and the expression of genes of interest indicates the changes relative to the control. The exact values of expression are above the bars. P-values ⩽ 0.05 were considered statistically significant where ⩽0.0332 (*), ⩽0.0021 (**), ⩽0.0002 (***), and ⩽0.0001 (****).

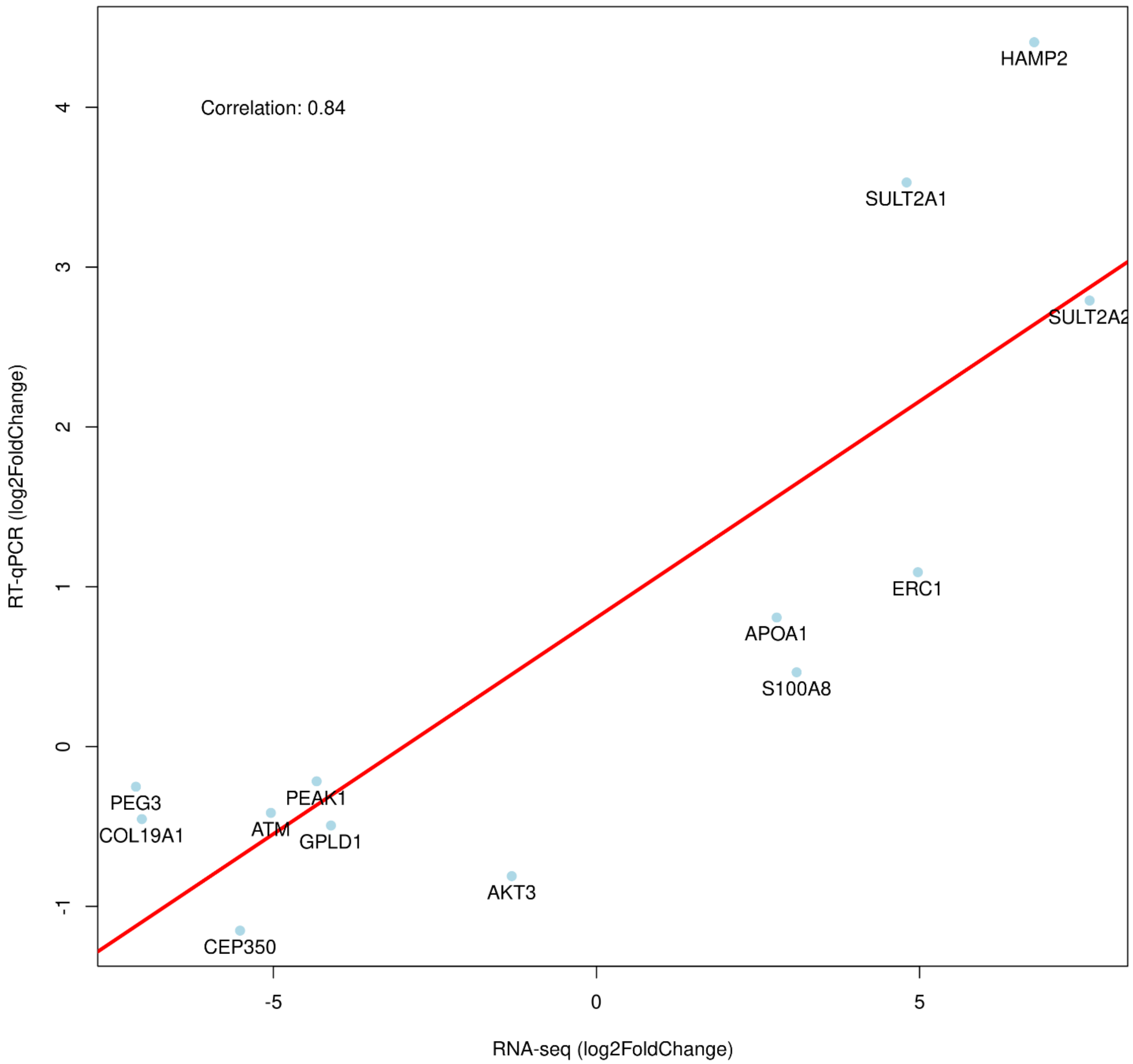

Supplementary Figure 8. Gene expression correlation between RT-qPCR and RNA-seq data. X-axis represents log2FoldChange od RNA-seq, Y-axis represents log2FoldChange of RT-qPCR for selected DEGs. The Pearson correlation coefficients and linear regression line are indicated.
